# Layered ZIFs Using a Surfactant as a Structure Directing Agent

**DOI:** 10.1021/jacs.5c18975

**Published:** 2026-02-09

**Authors:** Xuekui Duan, Shuqing Song, Céline Besnard, Pascal Alexander Schouwink, Yueqing Shen, Heng-Yu Chi, Jian Hao, Laura Piveteau, Kumar Varoon Agrawal

**Affiliations:** a Laboratory of Advanced Separations (LAS), École Polytechnique Fédérale de Lausanne (EPFL), Sion CH-1951, Switzerland; b Laboratoire de Cristallographie, Ecole de Physique, University of Geneva, 24 Quai E. Ansermet, Geneva 4 CH-1211, Switzerland; c Institut des Sciences et Ingénierie Chimiques (ISIC), École Polytechnique Fédérale de Lausanne (EPFL), Lausanne CH-1015, Switzerland

## Abstract

Zeolitic imidazolate frameworks (ZIFs) are three-dimensional (3D) porous materials with only a few exceptions - ZIF-L, Zn_2_(benzimidazole)_4_, etc. Herein, we report the synthesis of a new layered ZIF, which we call ZIF-S. We use a surfactant (sodium dodecyl sulfate) as a structure-directing agent, analogous to the concept used in the synthesis of zeolites. The layers contain individual ZIF sheets intercalated by surfactants. Its ordered structure belongs to the tetragonal lattice with the *P*4̅2_1_ *m* space group. The unit cell parameters are *a* = *b* = 16.82 Å; *c* = 24.5 Å at room temperature. The layered material undergoes topotactic condensation and forms its parent material (ZIF-8 or ZIF-67, depending on the metal node) upon heating to or above 200 °C. ZIF-S layers could be obtained with a large lateral size and a high aspect ratio, which is ideal for the scalable preparation of gas-selective membranes, thanks to the presence of pore apertures suitable for the separation of small gas molecules. Fabrication of gas-selective membranes from a simple coating of ZIF-S is demonstrated.

## Graphical abstract

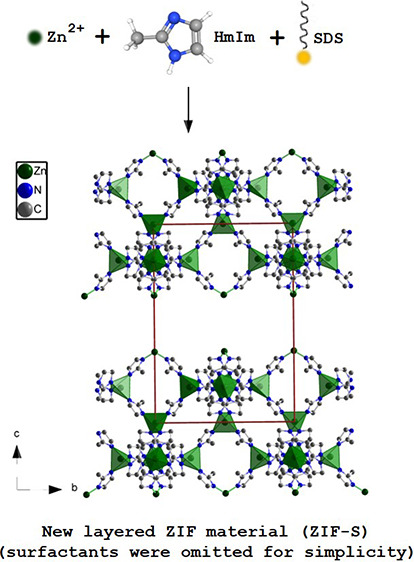

## Introduction

ZIFs represent an important class of porous materials belonging to the broader family of metal–organic frameworks (MOFs).^1^ ZIFs are often formed by using a transition metal node (e.g., zinc or cobalt) linked by imidazolate-based organic ligands. The metal ions are bridged tetrahedrally with a near 145° bond angle, mimicking the bonding arrangement of zeolites.^2^ The combination of coordination chemistry and zeolite topology endows ZIFs with unique properties, including tunable synthesis by a wide range of synthesis techniques,^3,4^ high surface area,^5,6^ high thermal stability,^7^ molecular selectivity,^8^ etc. These properties make ZIFs attractive in a variety of applications, e.g., molecular separation,^6,8–10^ drug delivery,^11^ sensing,^12^ and catalysis.^13,14^

ZIFs are traditionally prepared in 3D structures. One of the most studied ZIFs is ZIF-8, which adopts sodalite (SOD) topology. However, a few two-dimensional (2D) ZIFs with layered morphology have been reported. ZIF-L consists of two-dimensional (2D) layers, separated by coordinated and free 2-methylimidazolium (HmIm) ligands.^15^ Zn_2_(benzimidazole)_4_ ^16^ and Co_4_(benzimidazole)_16_ ^17^ consist of 2D layers held together by weak van der Waals interactions. Compared with 3D ZIFs, 2D ZIFs offer unique advantages from sheet-like morphology, e.g., a high surface-to-volume ratio, abundant surface-active sites, and the ability to be exfoliated to thin nanosheets. These properties are beneficial for the construction of ultrathin selective membranes,^16,18,19^ for the preparation of efficient adsorbents^20,21^ and drug delivery systems,^22^ and for producing ZIF-derived catalysts^23^ and electrodes.^24,25^

Despite the large number of ZIF structures, only a few have been reported in 2D layered architecture. This is due to the tetrahedral coordination environment of the metal node, which promotes structure propagation in three dimensions unless coordination can be somehow terminated. This is analogous to zeolites, which were traditionally viewed as 3D materials until the discovery of MCM-22,^26^ and many other 2D layered zeolites following it.^27,28^ Layered 2D zeolites could be synthesized using a long-chain surfactant as a structure-directing agent.^29^ We hypothesized that 2D layered ZIFs could also be obtained by using a surfactant-based templating approach. Lotsch et al. showed that a cationic surfactant, cetyltrimethylammonium bromide (CTAB), can be used to template mesostructured ZIFs.^30–32^ However, the synthesized materials either lacked a framework structure within the 2D layers^30,32^ or represented simply a mesostructure of ZIF layers and CTAB micelles connected by weak van der Waals interactions.^31^

Herein, we report a new layered ZIF that we refer to as ZIF-S. The layered morphology in ZIF-S is induced by the use of an anionic surfactant, sodium dodecyl sulfate (SDS), which acts as a structure-directing agent. Dodecyl sulfate ions terminate the metal-linker coordination by bonding with the terminal metal, facilitating the formation of 2D layers. The surfactant ion also separates the layers by intercalating the gallery space of the layers. In the literature, SDS has been used for the synthesis of ZIF-8/ZIF-67 nanoplatelets.^33^ However, in these nanoplatelets, the surfactant acts as a capping agent to inhibit out-of-plane crystal growth. As a result, the surfactant is not incorporated into the structure, providing a nonlayered morphology. In contrast, SDS in ZIF-S acts as an SDA, intercalating the layered space and holding together the ZIF layers, analogous to the function of SDA in the case of 2D zeolites.^34,27^ The layered structure was solved by using 3D electron diffraction (MicroED). Heating ZIF-S above 200 °C induced topotactic condensation into a 3D structure, as confirmed by in situ powder X-ray diffraction (XRD) measurements. Owing to the large lateral size and high aspect ratio of ZIF-S nanosheets, a simple vacuum filtration assembly could be used to fabricate molecular sieving membranes.

## Results and Discussion

The synthesis of ZIF-S was achieved using Zn or Co as the metal source, HmIm as the organic linker, and SDS as the SDA, in aqueous solutions and at room temperature. SDS has been previously used to form ZIF platelets; however, a layered structure has not been reported.^33^ As we show here, the concentration of the precursors plays a role in determining whether a layered structure will form. In the literature, the following composition of the growth solution is typical: 1 metal:8 HmIm:1.2 SDS with a metal concentration of ∼24 mM. This composition yields ZIF-8 (with Zn) or ZIF-67 (with Co). We decided to explore much lower concentrations of the precursor solution, given that a lower concentration will reduce the nucleation kinetics for the 3D ZIF phase as indicated by our recent study.^35^

Figure S1 shows the scanning electron microscopy (SEM) and XRD of the three materials synthesized with a Co^2+^ concentration of 2 mM and a Co/HmIm ratio of 1/8 and with three SDS concentrations of 0.5, 1, and 2 mM. We obtained three different materials with unknown structures. We then sought to increase the HmIm/Co ratio from 8 to 72, which is typical for yielding ZIF-67. We used molar concentrations of Co/HmIm/SDS = 1.35 mM:97.44 mM:1.54 mM. This dilute precursor solution with a higher HmIm/Co ratio yielded the new ZIF-S material, as shown in Figure S2a, with well-faceted square morphology. We then tried to reduce the amount of SDS in the system, from 1.54 to 0.77 mM, then to 0.385 mM. When the SDS amount was reduced to 0.77 mM, we still obtained thin and well-faceted ZIF-S square crystals (Figure S2b). When the SDS amount was reduced to 0.385, a ZIF-67 phase was obtained with some unclear impurity phases (Figure S2c). Therefore, the optimal composition was Co/HmIm/SDS = 1.35 mM:97.44 mM:0.77 mM.

Next, we studied the growth process of ZIF-S at room temperature. Since the Zn-HmIm system has slower kinetics than the Co-HmIm system, we first studied the growth of ZIF-S-Zn. The SEM images in Figure S3 show crystallization progressing as a function of synthesis time. At a short reaction time (1 h, Figure S3a), only aggregates could be observed from the synthesis solution. When the reaction time was increased to 2 h (Figure S3b), nanosheets with a square morphology started to form. When the reaction time was increased to 4 h (Figure S3c), nanosheets acquired well-faceted square morphology. Therefore, the optimum synthesis time was 4 h.

Detailed characterizations were performed on ZIF-S-Zn synthesized in 4 h. The SEM and TEM images of ZIF-S-Zn nanosheets reveal a well-faceted square morphology (Figure 1a, b). Selected area electron diffraction (SAED) patterns revealed a square 2D lattice in the in-plane direction (Figure 1c). Powder XRD confirmed that the material is distinct from that of ZIF-8 (Figure 1d). The presence of a diffraction peak at a low angle (2θ = 3.6°) suggests that the ZIF-S is likely layered. Fourier-transform infrared (FTIR) spectroscopy (Figure 1e) also revealed a spectrum distinct from that of ZIF-8, with additional vibration modes appearing at wavenumbers of 2922.4, 2852.9, 1246.9, 1206.5, 622.8, and 583.5 cm^–1^. We compared the FTIR spectrum of ZIF-S with that of SDS. The comparison revealed that the additional vibrational modes correspond to stretching modes of SDS. Peaks at 2922.4 and 2852.9 cm^–1^ correspond to the stretching of the hydrophobic tails, and the peaks at 1246.9, 1206.5, 622.8, and 583.5 cm^–1^ correspond to the stretching of the −SO_3_–R head groups.^36,37^ This implies that the surfactant chains were incorporated into the structure. Inductively coupled plasma mass spectrometry (ICP-MS) analysis revealed that ZIF-S does not contain Na. This suggests that the anionic surfactant chain is likely coordinated in the structure and extra-framework SDS is not present in the structure.

**Figure 1:**
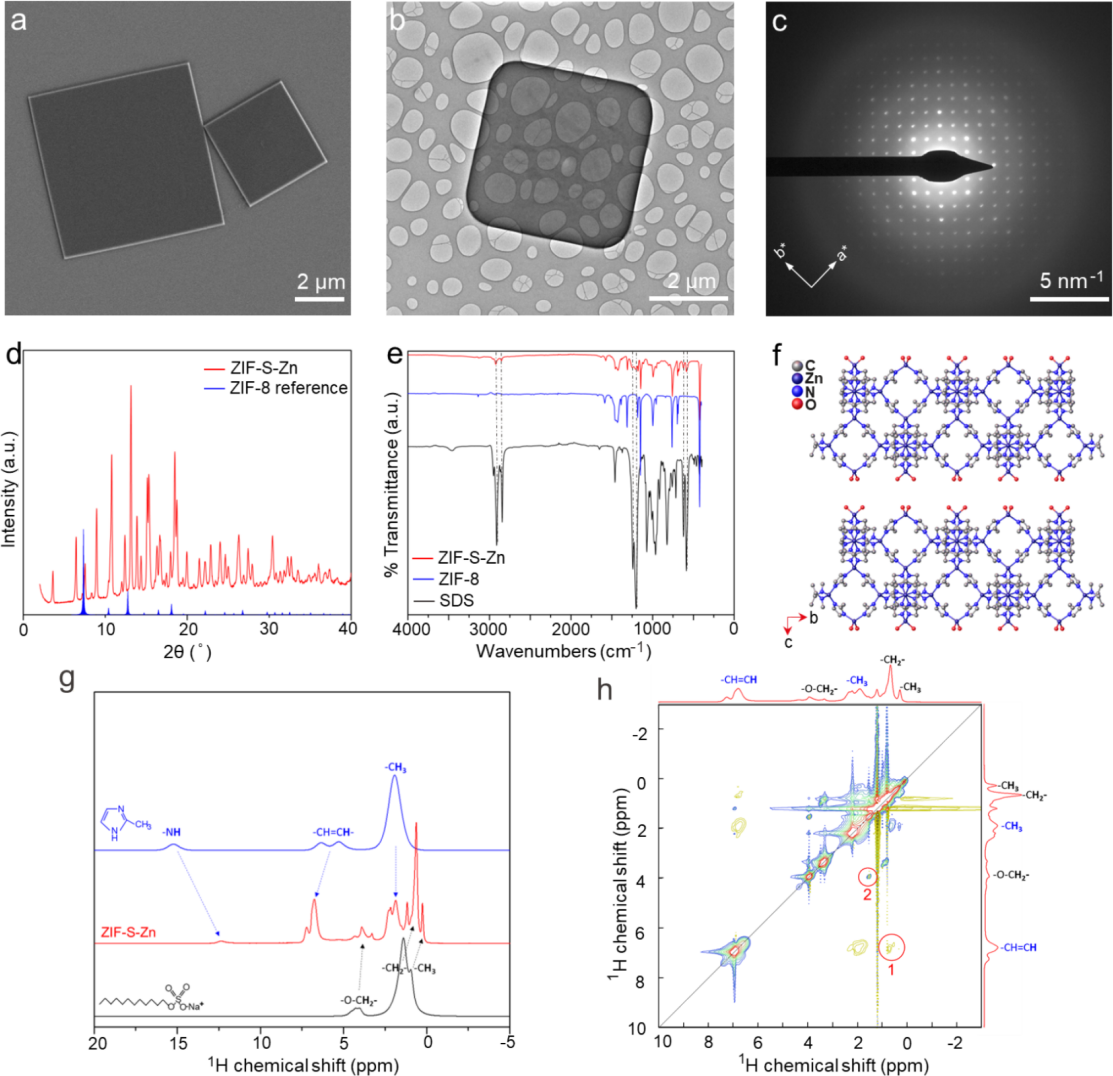
Characterization of ZIF-S-Zn synthesized at room temperature in 4 h. (a) SEM image showing the typical morphology and size of the synthesized nanosheets; (b) TEM image showing the well-faceted square morphology of the nanosheet; (c) the corresponding SAED pattern of the nanoplatelet shown in (b); (d) XRD analysis of the ZIF-S-Zn powder; (e) FTIR spectrum of the layered ZIF-S-Zn material and its comparison with that of ZIF-8 and SDS; (f) structure model of the layered ZIF-S-Zn material solved by the microED technique; (g) comparison of ^1^H solid-state NMR spectra of the ZIF-S-Zn material with that of the two precursors (HmIm and SDS); (h) ^1^H-^1^H EXSY solid-state NMR spectrum of the layered ZIF-S-Zn material.

3D electron diffraction was used to resolve the structure of ZIF-S. The structure could be solved using ShelXD^38^ in the space group *P*4̅2_1_ *m*, which confirmed a layered structure. The structure was refined in ShelXL^39^ using the kinematical scattering approximation. Geometrical restraints to the ligands were added to regularize the geometry as well as restraints on the displacement parameters. Due to the limited resolution of the data, likely due to disorder in the intercalated surfactant, only the layers could be modeled. The asymmetric unit of the model consists of three Zn atoms, two complete ligand molecules, and two half ligand molecules, which yields a chemical formula of Zn_1.75_(N_2_C_4_H_5_)_3_. This formula has a charge deficiency. There should be an added fragment corresponding to half of a negative charge per asymmetric unit. The chemical nature of this added charge could not be determined unambiguously from the microED data. However, from the material’s chemistry point of view, the positively charged ZIF layers would be balanced by coordination with negatively charged dodecyl sulfate ions. This is also supported by ICP analysis, which confirms that Na is not present in the structure. Therefore, dodecyl sulfate ions must be balanced by Zn metal centers. Accordingly, we assign the formula of ZIF-S as Zn_1.75_(N_2_C_4_H_5_)_3_(C_12_H_25_SO_4_)_0.5_. The unit cell parameters of the material were determined to be *a* = *b* = 16.43 Å; *c* = 23.4 Å at 100 K and *a* = *b* = 16.82 Å; *c* = 24.5 Å at room temperature. A view of the structure along the *a-*out-of-plane direction is shown in Figure 1f. Surfactant chains are not shown for the sake of simplicity. The structure consists of single ZIF-8 layers intercalated by the surfactant chains along the *c*-out-of-plane direction. Although the precise position of the surfactant chains could not be determined due to limited data and likely due to disorder of the surfactant, they should occupy the interlayer spacing and intercalate the ZIF layers. This is further corroborated by direct space modeling of the XRD data, where inclusion and optimization of the surfactant molecule in the model improve the fit significantly (Figure S4). These attempts in modeling the powder XRD data were not used as the basis of discussion here since the orientation of the surfactant cannot be unambiguously determined.

Solid-state nuclear magnetic resonance (NMR) measurements also provided structural information on the ZIF-S material. Figure 1g shows the ^1^H solid-state NMR spectra of the ZIF-S-Zn material as compared to those of the two precursors (HmIm and SDS). Based on the proton and carbon chemical shifts and their correlation in the ^1^H-^13^C heteronuclear correlation (HETCOR) spectrum (Figure S5), we could assign the peaks as illustrated in Figure 1g. The material exhibited a combination of peaks from both HmIm and the surfactant, indicating that the surfactant chains were incorporated into the structure. It is noted that the ^1^H NMR spectrum for ZIF-S-Zn contains a −NH peak, which should not be a part of the ZIF material. Rather, it should come from unreacted HmIm that was not washed out thoroughly. This was due to the fact that the ZIF-S could not be washed by solvent, otherwise it would either be dissolved (if using water, Figure S6) or transform to ZIF-8 (if using ethanol, Figure S7). Moreover, a ^1^H-^1^H exchange spectroscopy (EXSY) solid-state NMR spectrum (Figure 1h) provided further information regarding the spatial proximities of the surfactant and the linker incorporated in the framework. As depicted in Figure 1h, correlation 1 suggested that the end group of the surfactant is a close neighbor of the imidazolium ring of the framework. Correlation 2 also suggested that the methyl group of the framework is in close proximity to the headgroup of the surfactant. These results are consistent with the structure model and the expected charge-balancing electrostatic interactions between the dodecyl sulfate and the ZIF framework layer.

Layered zeolite precursors can be used to synthesize 3D frameworks by topotactic condensation.^40–43^ The same has been observed for ZIF-L.^44^ Similarly, ZIF-S also underwent topotactic condensation upon heat treatment. Figure 2a illustrates the phase change by the topotactic condensation. The material maintained its layered structure at temperatures below 180 °C. Upon heating to 200 °C, the surfactant chains would decompose, and the ZIF layers would condense to form the ZIF-8 structure, as evidenced by the in situ XRD patterns measured at 200 and 250 °C (Figure 2b). The thermogravimetric (TGA) (Figure S9) and differential scanning calorimetry (DSC) (Figure 2c) studies also suggested that the surfactant started to decompose at temperatures around 180–200 °C. This is in contrast with the previously reported CTAB templated MIF-1 material, which did not show a thermally induced phase transition. Rather, MIF-1 converted to mesostructure lacking long-range order upon heating.^30^ Moreover, ZIF-S-Zn also transforms to ZIF-8 upon ethanol washing at room temperature (Figure S7). We also performed Ar adsorption/desorption measurements on the as-made ZIF-S material as well as the heat-treated sample (200 °C in air) and the ethanol-washed sample (Figure 2d). The as-synthesized ZIF-S material had a very small specific surface area of only 4.489 m^2^/g. After heat treatment, the surface area increased slightly to 10.235 m^2^/g. This indicated that heat treatment at 200 °C cannot fully remove the surfactant, consistent with the XRD study. Ethanol washing is more effective in removing the occluded surfactant, resulting in a BET specific surface area of 631.2 m^2^/g (Figure 2e).

**Figure 2:**
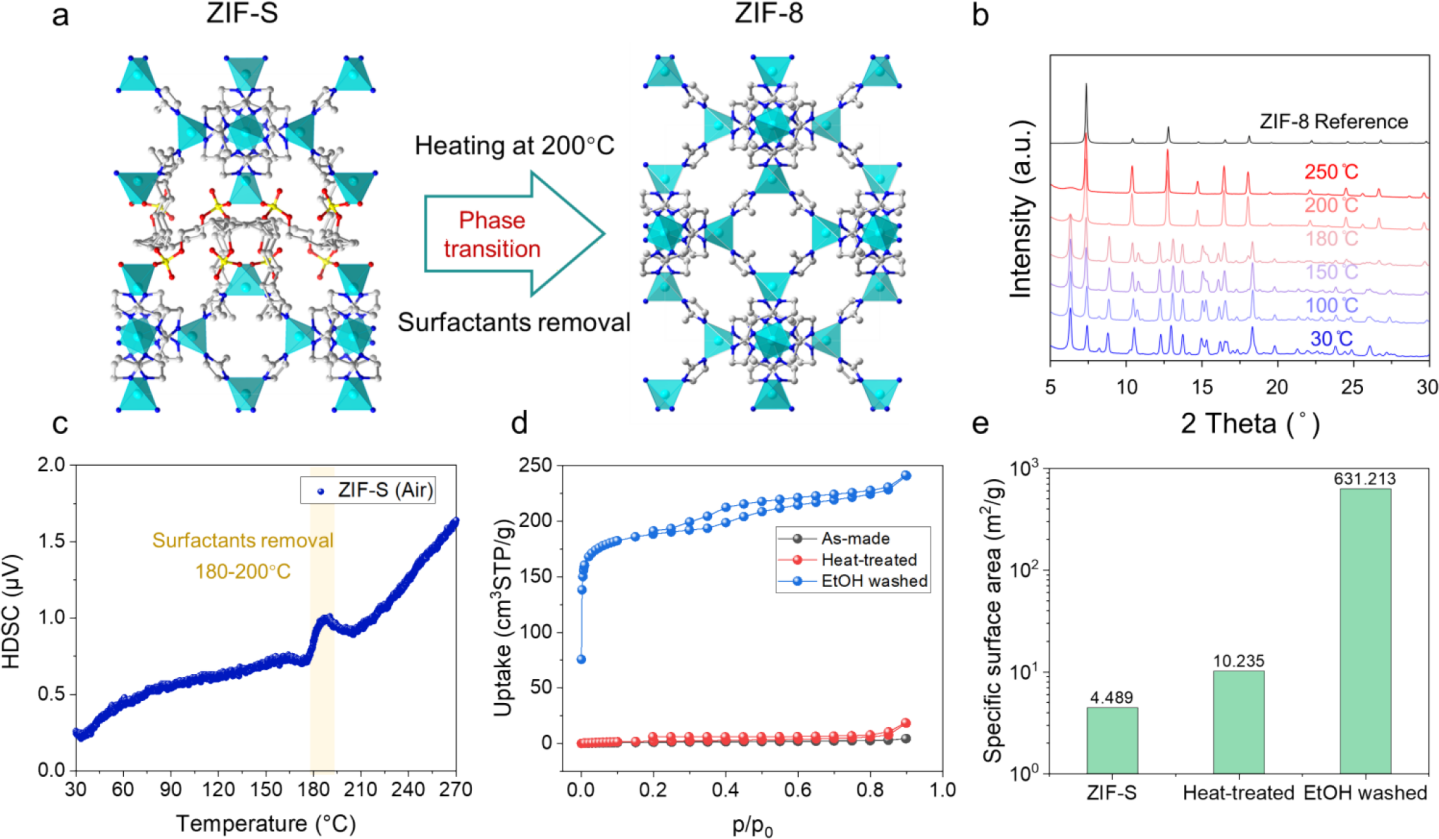
(a) The structure model of the layered ZIF-S-Zn material viewed along the *a*-axis, as well as the structure model of ZIF-8 material viewed along the *a*-axis, illustrating the topotactic condensation of the layered ZIF-S-Zn to ZIF-8; (b) powder XRD patterns of the layered ZIF-S-Zn material at different temperatures. The data were generated by in situ heating while measuring XRD; (c) differential scanning calorimetry (DSC) measurement of the ZIF-S material showing the transition; (d) Ar adsorption/desorption (87 K) measurement of the as-made ZIF-S material as well as the heat-treated ZIF-S material (200 °C in air for 8 h) and ethanol-washed ZIF-S material; (e) comparison of the BET specific surface areas of as-made, heat-treated (200 °C, 8 h), and ethanol washed ZIF-S.

The as-synthesized layered ZIF-S-Zn, possessing a large lateral size and a high aspect ratio, is attractive as a building block for thin membranes. They were used to deposit thin films on a porous polybenzimidazole (PBI) support^45^ by a simple vacuum filtration method.^46,47^ Before vacuum filtration, the suspension was centrifuged at 10000 rpm for 10 min to remove relatively thick layered platelets. Then, the suspension was kept static for 24 h to allow the remaining thicker platelets to settle. These settled platelets had a thickness in the range of 40–60 nm (Figure 3a, c1, Figure S10). We noticed that the nanoplatelets have a spiral morphology (Figure 3a, Figure S10). This indicates that the crystals were grown by the well-established spiral crystal growth mechanism.^48^ After sedimentation, only mostly the thinner platelets, referred to here as ZIF-S-Zn nanosheets, with a thickness of approximately 10 nm (Figure 3b, c2, Figure S10), remained in the suspension and were then used to deposit thin films. The PBI supports, prepared by nonsolvent-induced phase separation, host a smooth surface and an average pore size of approximately 20 nm (Figure 3d). The chemical compatibility of PBI and the HmIm groups of the nanosheets makes these porous supports ideal for nanosheet deposition. Upon filtration, the ZIF-S-Zn nanosheets were deposited in an oriented fashion, leading to a continuous thin film. Figure 3e shows the surface morphology of the as-prepared ZIF-S-Zn film after filtration, demonstrating that a compact, defect-free film was successfully prepared.

**Figure 3:**
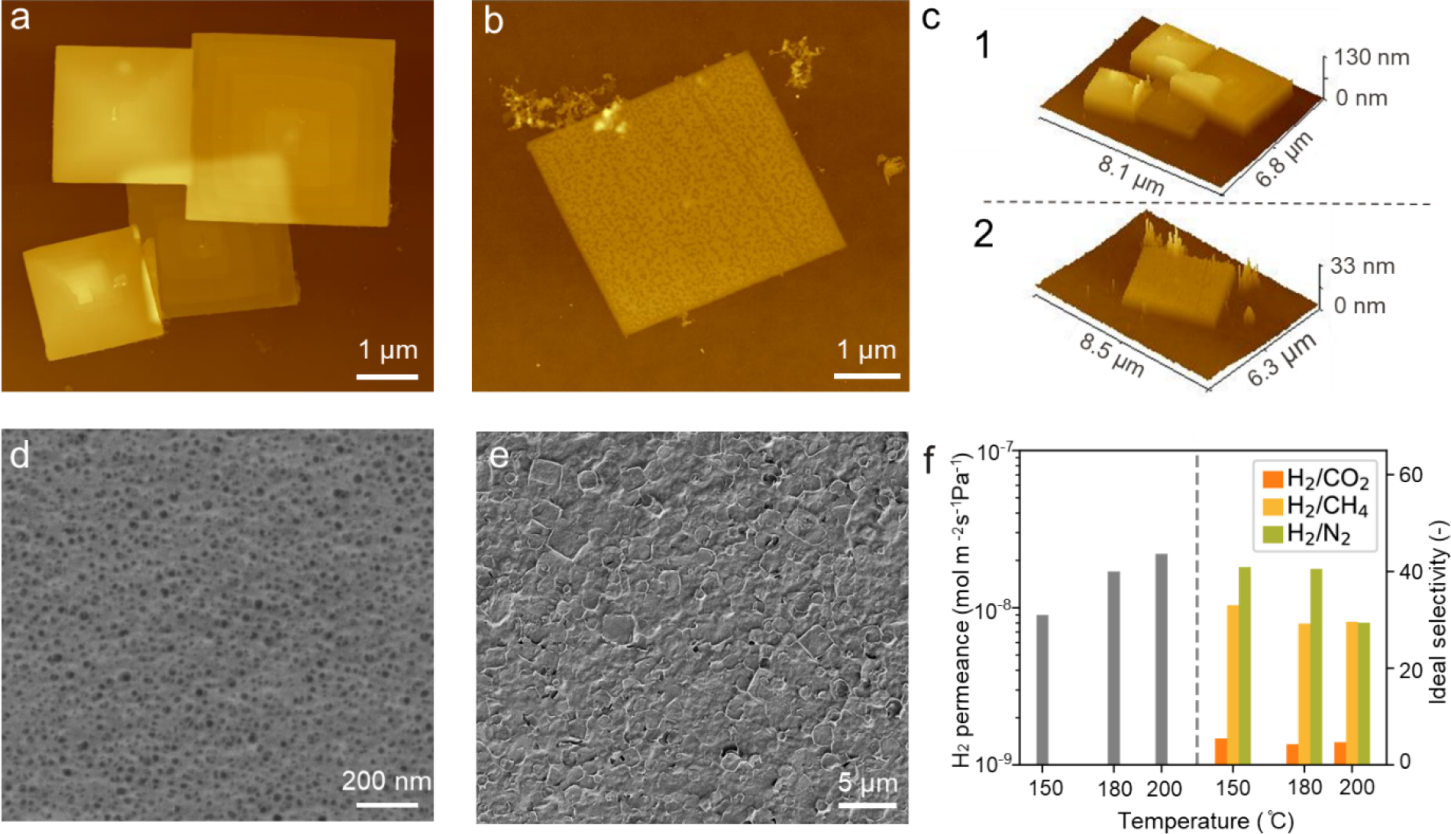
AFM image of the ZIF-S-Zn nanoplatelets after centrifugation at 10000 rpm for 10 min (a) and nanosheets after sedimentation for 24 h (b); (c) height profiles of the nanoplatelets/nanosheet shown in (a) and (b); (d) SEM image of the porous PBI support; (e) top-view SEM image of the ZIF-S-Zn membrane; (f) single-gas permeation results showing H_2_ permeance as a function of temperature, and the corresponding H_2_/CO_2_, H_2_/CH_4_, and H_2_/N_2_ ideal selectivities. Measurements were conducted using a pure gas feed (H_2_, CO_2_, CH_4_, N_2_) at 2 bar on the feed side and Ar as sweep gas at 1 bar on the permeate side.

Single gas permeation tests were performed on the as-prepared ZIF-S-Zn nanosheet membrane to examine its molecular sieving properties. At room temperature, the membrane yielded an extremely low gas permeance (10^–12^ mol m^–2^ s^–1^ Pa^–1^). This is likely due to blockage of the small 4-membered-ring (4-MR) pore aperture by the surfactant chains in the gallery. Under such conditions, gas transport is typically activated. Consequently, transport was measured at elevated temperatures. At 150 °C, the H_2_ permeance increased by nearly four orders of magnitude to 9 × 10^–9^ mol m^–2^ s^–1^ Pa^–1^. The ideal selectivities of H_2_ with respect to CH_4_ and N_2_ were 33 and 40, respectively. The H_2_/CO_2_ ideal selectivity was 5. This suggests that the effective pore size is larger than the kinetic diameter of CO_2_ (3.3 Å) but smaller than those of CH_4_ (3.8 Å) and N_2_ (3.64 Å). Upon further increasing the temperature to 200 °C, the membrane showed an increased H_2_ permeance of 2.2 × 10^–8^ mol m^–2^ s^–1^ Pa^–1^ with ideal selectivities of H_2_ with respect to N_2_ and CH_4_ of 29.3 and 30, respectively. The relatively low gas permeance (even after activation at 200 °C) was attributed to the fact that thermal activation is not fully effective to remove the occluded surfactant. This is supported by our Ar adsorption/desorption measurement (Figure 2d).

H_2_/CO_2_ selective membranes are attractive for precombustion carbon capture.^49^ We sought to study an isostructure of ZIF-S-Zn by replacing the Zn nodes with Co, analogous to the case of ZIF-8 and ZIF-67. Literature shows that ZIF-67 has a more rigid Co-HmIm bond than the Zn-HmIm bond.^50,51^ Based on this, we anticipated that ZIF-S-Co would demonstrate higher H_2_/CO_2_ selectivity than ZIF-S-Zn. Preparation of ZIF-S-Co nanosheets followed a similar recipe to the ZIF-S-Zn case, except that Zn­(NO_3_)_2_·6H_2_O was replaced by Co­(NO_3_)_2_·6H_2_O, and the synthesis time was reduced from 4 to 1 h, thanks to a higher crystallization kinetics of the Co-HmIm system. The as-prepared ZIF-S-Co nanosheets had a morphology corresponding to a sheet with a square morphology (Figure 4a and b). SAED revealed a square 2D lattice in the in-plane direction (Figure 4c). The XRD and FTIR spectra confirmed that the structure is identical to that of the layered ZIF-S-Zn (Figure 4d and e). The layered ZIF-S-Co material also underwent topotactic condensation upon heat treatment above 200 °C, changing from its layered structure to the ZIF-67 structure, as confirmed by the in situ XRD experiments (Figure 4f).

**Figure 4:**
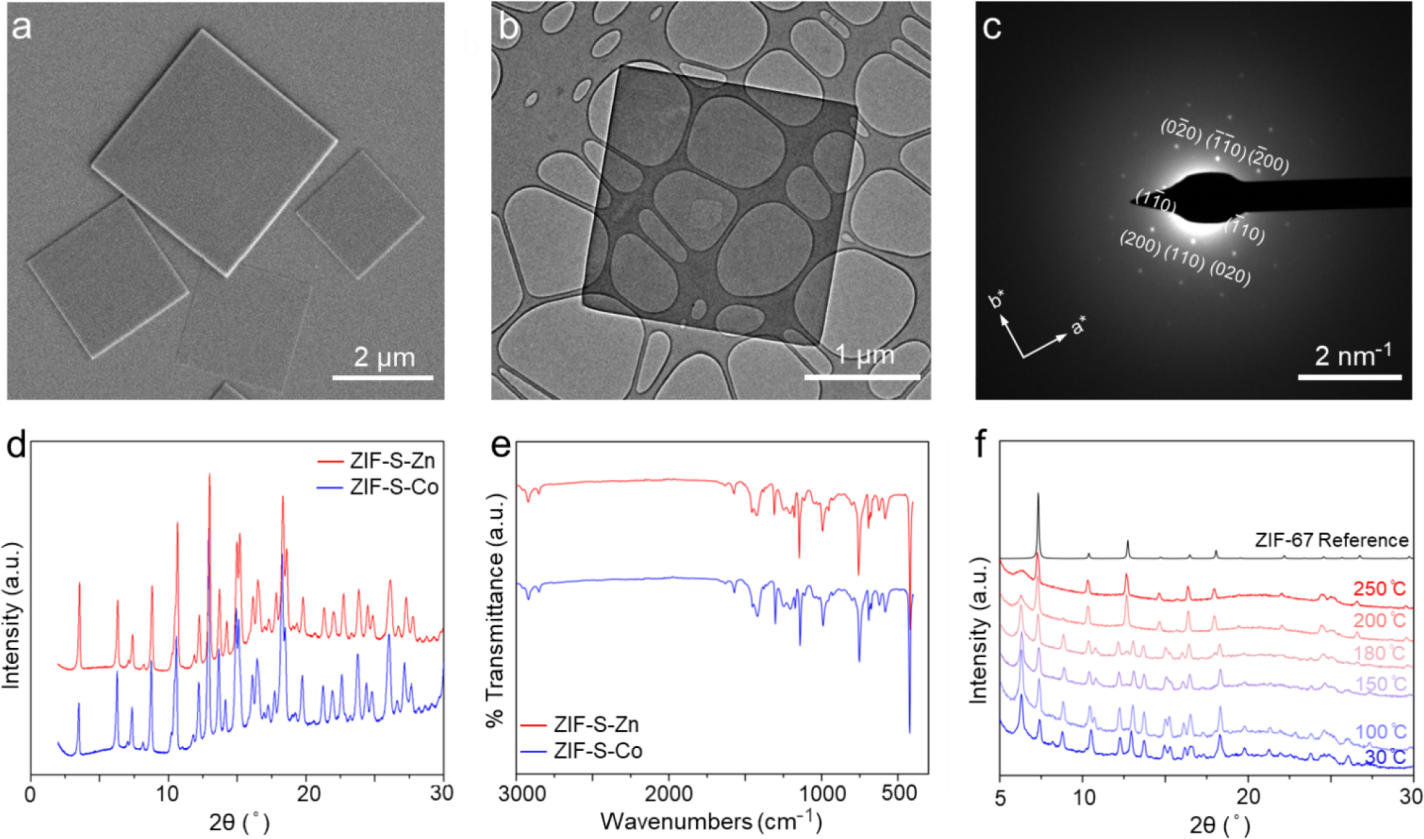
Characterization of the layered ZIF-S-Co material synthesized at room temperature for 1 h: (a) SEM image showing the typical morphology and size of the synthesized nanosheets; (b) TEM image showing the well-faceted square morphology of the nanosheet; (c) the corresponding SAED pattern of the nanoplatelet shown in (b); (d) powder XRD pattern of the ZIF-S-Co and its comparison with that of the ZIF-S-Zn; (e) FTIR spectra of the ZIF-S-Co and its comparison with that of the ZIF-S-Zn; (f) powder XRD patterns of ZIF-S-Co at different temperatures. The data was generated by in situ heating while measuring XRD.

Using the same membrane preparation protocol as was used for the ZIF-S-Zn case, compact and continuous ZIF-S-Co deposits could also be obtained. Figure 5a shows the AFM image of the thicker ZIF-S-Co platelets after centrifugation at 10000 rpm for 10 min. They had thicknesses of 60–80 nm (Figure 5c1, Figure S11). The solution was kept static for 24 h to remove most of the thicker platelets by sedimentation. Only thinner nanosheets, with a thickness of ∼5–20 nm (Figure 5b, c2, and Figure S11), were used for membrane preparation. Figure 5d shows a typical SEM image of the as-prepared ZIF-S-Co membrane. The membrane appeared compact and continuous, with a thickness of approximately 300 nm as measured by cross-section prepared by a focused ion beam (FIB, Figure 5e).

**Figure 5:**
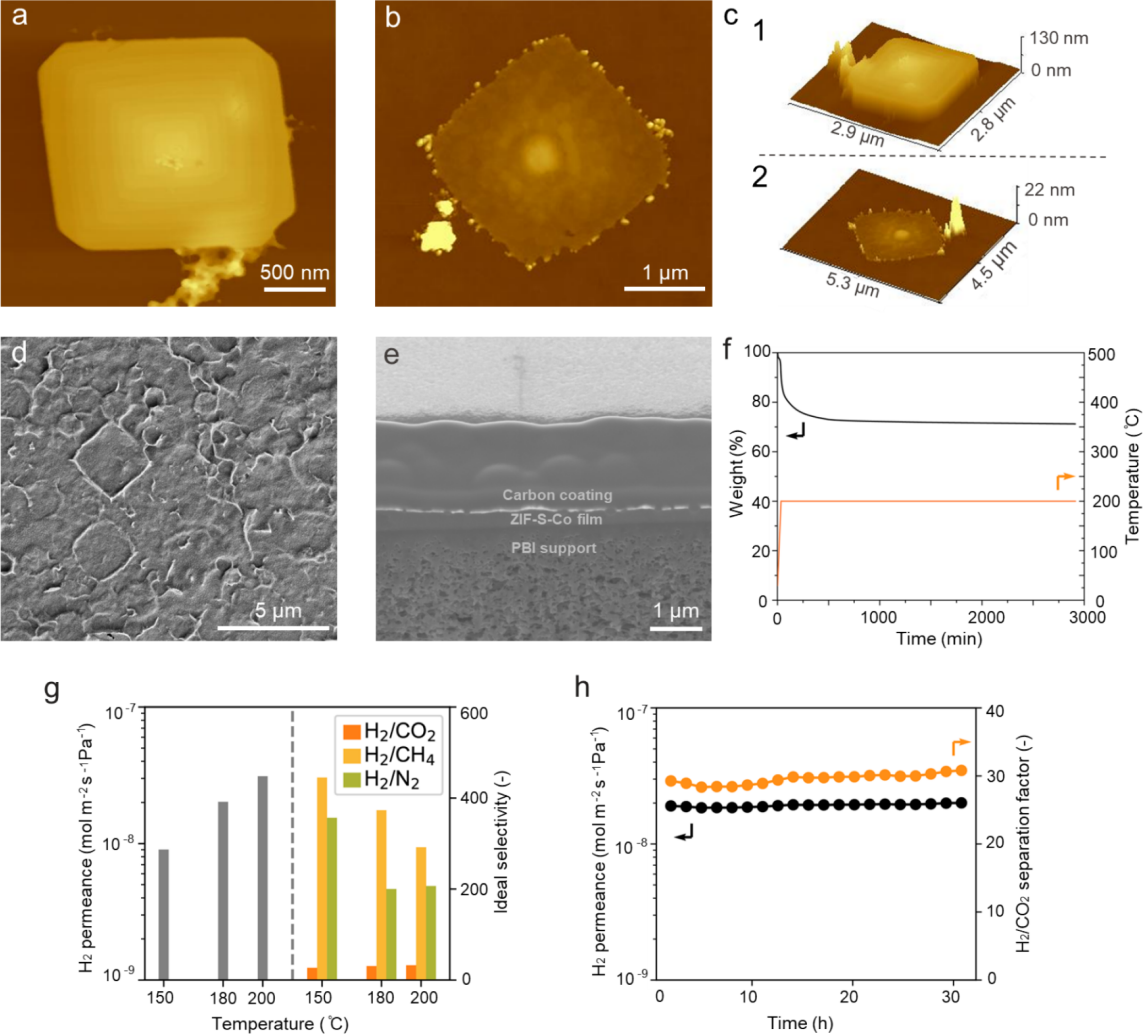
(a) AFM image of the ZIF-S-Co nanoplatelet after centrifuging at 10,000 rpm for 10 min; (b) AFM image of the ZIF-S-Co nanosheets after centrifugation at 10,000 rpm for 10 min followed by sedimentation for 24 h; (c) the height profiles of the nanoplatelet/nanosheet shown in (a) and (d); (d) top-view SEM image of the prepared ZIF-S-Co membrane; (e) single gas permeation results showing H_2_ permeance as a function of testing temperature and the corresponding H_2_/CO_2_, H_2_/CH_4_, and H_2_/N_2_ ideal selectivities. Measurements were conducted using a pure gas feed (H_2_, CO_2_, CH_4_, N_2_) at 2 bar on the feed side and Ar as sweep gas at 1 bar on the permeate side; (g) stability test of the ZIF-S-Co material in humid N_2_ (∼2.5 mol % water vapor) at 200 °C probed by TGA showing no mass loss at 200 °C for prolonged time even at a humid atmosphere; (h) membrane stability test using a 50/50 mol % H_2_/CO_2_ gas mixture with ∼1.5 mol % water vapor at 200 °C. The feed mixture was kept at 2 bar, while the permeate was an Ar sweep at 1 bar.

Single gas permeation tests from ZIF-Zn-Co membranes showed a trend similar to that of the case of the ZIF-S-Zn membrane (Figure 5g). At room temperature, the membrane had a low gas permeance due to the blockage of 4-MR by the surfactant chains. The membrane also showed increased gas transport with increasing temperature. For example, H_2_ permeance increased to 9 × 10^–9^ mol m^–2^ s^–1^ Pa^–1^ at 150 °C and continued to increase to 2 × 10^–8^ mol m^–2^ s^–1^ Pa^–1^ at 180 °C and 3.1 × 10^–8^ mol m^–2^ s^–1^ Pa^–1^ at 200 °C. The ideal selectivities of H_2_ over CO_2_, CH_4_, and N_2_ were close to 30, 400, and 200, respectively. This proves that the ZIF-S-Co membrane has more rigid 4-MR openings compared with the ZIF-S-Zn membrane. The effective pore opening in this case lies between the kinetic diameter of H_2_ (2.89 Å) and CO_2_ (3.3 Å), making the membrane selective for H_2_ over CO_2_.

One consideration of membranes for precombustion carbon capture is their stability under high-temperature water vapor. To study the stability of our ZIF-S-Co membranes under water vapor at high temperature, we first carried out a TGA test under a humidified atmosphere (∼2.5 mol %). Figure 5f shows that at 200 °C, the material is stable for the tested period (2 days). The weight loss at 200 °C corresponds to the removal of the surfactant chains. We further demonstrated the stability of our membrane under a humidified atmosphere by a long-term membrane separation test at 200 °C using a 50/50 mol % H_2_/CO_2_ mixture with ∼1.5 mol % water vapor. Figure 5h demonstrates that the membrane performed consistently, even with the presence of water vapor. It showed a stable H_2_ permeance of 3.1 × 10^–8^ mol m^–2^ s^–1^ Pa^–1^ and a corresponding H_2_/CO_2_ separation factor close to 30.

The above membrane fabrication methods represent only two examples of how the ZIF-S nanosheets could be used for making membranes. In fact, ZIF-S provides a versatile platform for facile membrane manufacturing. Another example is given below. Taking advantage of the fact that the surfactant within ZIF-S layers could be easily removed by ethanol washing (Figure S7), one could use an in situ ethanol washing to activate the layers after filter-deposited on a PBI support. This in situ washing step would generate defects in the as-filtered film (Figure S13). However, a secondary growth procedure using a Co/HmIm of 4 mM:32 mM precursor solution for 12 h at room temperature (see SI Note S1 for details) resulted in gas-selective membranes. Figure S13 shows the SEM images and XRD patterns of the as-filtered ZIF-S-Co film and the films after ethanol washing and after secondary growth. XRD measurements confirmed that ethanol washing was effective in removing the surfactant, and the ZIF-S-Co film was transformed to ZIF-67. The ZIF-67 film was highly oriented, along the *c-*out-of-plane direction, thanks to the horizontal stacking of the nanosheets on PBI supports. After secondary growth, the membrane showed a high H_2_ permeance of 6.7 × 10^–7^ mol m^–2^ s^–1^ Pa^–1^ and corresponding H_2_/CO_2_ separation factor of 22 at 150 °C. The higher H_2_ permeance was attributed to the fact that ethanol washing was able to remove most of the surfactant more effectively.

## Conclusions

We demonstrated the synthesis of a new layered ZIF structure (ZIF-S) templated by surfactant chains (dodecyl sulfate ions). This material could be synthesized in both the Zn form and the Co form, depending on the metal source used. The crystal structure of the material was solved by the microED technique, showing single ZIF-8/ZIF-67 sheets intercalated by surfactant chains. Owing to the nanosheet morphology of ZIF-S material, facile vacuum filtration on porous supports could be used to prepare high-quality membranes. Several routes for membrane fabrication were demonstrated. In particular, ZIF-S-Co resulted in membranes with large gas pair selectivities. Overall, this report sheds light on the synthesis of a new layered ZIF/MOF structure using a surfactant as the SDA. This method reported here can potentially be used to develop other novel layered ZIF/MOF, with applications not limited to membrane separation but also catalysis, sensing, energy storage, electronic devices, drug delivery, and other nanomaterial-based technologies.

## Supplementary Material



## Notes

Additional experimental methods, materials, and details; additional supporting data including SEM, structure model, solid-state NMR, PXRD, TGA, AFM, gas permeation data (PDF)

## References

[1] Tan Y. X., Wang F., Zhang J. (2018). Design and synthesis of multifunctional
metal-organic zeolites. Chem. Soc. Rev..

[2] Banerjee R., Phan A., Wang B., Knobler C., Furukawa H., O’Keeffe M., Yaghi O. M. (2008). High-throughput synthesis of zeolitic
imidazolate frameworks and application to CO2 capture. Science.

[3] Chen B., Yang Z., Zhu Y., Xia Y. (2014). Zeolitic imidazolate
framework materials: recent progress in synthesis and applications. J. Mater. Chem. A.

[4] Kouser S., Hezam A., Khadri M. N., Khanum S. A. (2022). A review on zeolite
imidazole frameworks: synthesis, properties, and applications. J. Porous Mater..

[5] Banerjee R., Furukawa H., Britt D., Knobler C., O’Keeffe M., Yaghi O. M. (2009). Control of pore
size and functionality in isoreticular
zeolitic imidazolate frameworks and their carbon dioxide selective
capture properties. J. Am. Chem. Soc..

[6] Ighalo J. O., Rangabhashiyam S., Adeyanju C. A., Ogunniyi S., Adeniyi A. G., Igwegbe C. A. (2022). Zeolitic
imidazolate frameworks (ZIFs) for aqueous
phase adsorption-a review. J. Ind. Eng. Chem..

[7] Healy C., Patil K. M., Wilson B. H., Hermanspahn L., Harvey-Reid N. C., Howard B. I., Kleinjan C., Kolien J., Payet F., Telfer S. G., Kruger P. E., Bennett T. D. (2020). The thermal
stability of metal-organic frameworks. Coord.
Chem. Rev..

[8] Pimentel B. R., Parulkar A., Zhou E. K., Brunelli N. A., Lively R. P. (2014). Zeolitic
imidazolate frameworks: next-generation materials for energy-efficient
gas separations. ChemSusChem.

[9] Wang B., Côté A. P., Furukawa H., O’Keeffe M., Yaghi O. M. (2008). Colossal cages in
zeolitic imidazolate frameworks as
selective carbon dioxide reservoirs. Nature.

[10] Ma X., Kumar P., Mittal N., Khlyustova A., Daoutidis P., Mkhoyan K. A., Tsapatsis M. (2018). Zeolitic imidazolate
framework membranes made by ligand-induced permselectivation. Science.

[11] Akhtar H., Amara U., Mahmood K., Hanif M., Khalid M., Qadir S., Peng Q., Safdar M., Amjad M., Saif M. Z., Tahir A., Yaqub M., Khalid K. (2024). Drug carrier
wonders: Synthetic strategies of zeolitic imidazolates frameworks
(ZIFs) and their applications in drug delivery and anti-cancer activity. Adv. Colloid Interface Sci..

[12] Zhang J., Tan Y., Song W. J. (2020). Zeolitic
imidazolate frameworks for use in electrochemical
and optical chemical sensing and biosensing: a review. Microchim. Acta.

[13] Duan C., Li F., Luo S., Xiao J., Li L., Xi H. (2018). Facile synthesis
of hierarchical porous metal-organic frameworks with enhanced catalytic
activity. Chem. Eng. J..

[14] Bhattacharjee S., Jang M. S., Kwon H. J., Ahn W. S. (2014). Zeolitic imidazolate
frameworks: synthesis, functionalization, and catalytic/adsorption
applications. Catal. Surv. Asia..

[15] Chen R., Yao J., Gu Q., Smeets S., Baerlocher C., Gu H., Zhu D., Morris W., Yaghi O. M., Wang H. (2013). A two-dimensional
zeolitic imidazolate framework with a cushion-shaped cavity for CO
2 adsorption. Chem. Commun..

[16] Peng Y., Li Y., Ban Y., Jin H., Jiao W., Liu X., Yang W. (2014). Metal-organic framework
nanosheets as building blocks for molecular
sieving membranes. Science.

[17] Jayaramulu K., Masa J., Morales D. M., Tomanec O., Ranc V., Petr M., Wilde P., Chen Y., Zboril R., Schuhmann W., Fischer R. A. (2018). Ultrathin 2D Cobalt Zeolite-Imidazole
Framework Nanosheets for Electrocatalytic Oxygen Evolution. Adv. Sci..

[18] Yu C., Kim Y. j., Kim J., Hayashi M., Kim D. W., Kwon H. T., Eum K. (2022). A sacrificial ZIF-L seed layer for
sub-100 nm thin propylene-selective ZIF-8 membranes. J. Mater. Chem. A.

[19] Jia Y., Yang J., Liu P., Chen K., Li J., Pi X., Han C., Zhang Y. (2024). Mixed matrix membranes containing
oriented two-dimensional ZIF-L nanosheets for efficient H_2_/CO_2_ separation. Sep. Purif. Technol..

[20] Nasir A. M., Md Nordin N. A. H., Goh P. S., Ismail A. F. (2018). Application of two-dimensional
leaf-shaped zeolitic imidazolate framework (2D ZIF-L) as arsenite
adsorbent: Kinetic, isotherm and mechanism. J. Mol. Liq..

[21] Huang C., Zhang H., Zheng K., Zhang Z., Jiang Q., Li J. (2021). Two-dimensional hydrophilic
ZIF-L as a highly-selective adsorbent
for rapid phosphate removal from wastewater. Sci. Total Environ..

[22] Song Y., Yang J., Wang L., Xie Z. (2020). Metal-Organic Sheets
for Efficient Drug Delivery and Bioimaging. ChemMedChem..

[23] Sun Q., Zhu G., Dai L., Meng W., Wang L., Liu S. (2023). High-Efficiency
Two-Dimensional Catalysts Derived from Co_x_Zn_y_-ZIF-L MOFs for Solid-State Na-Air Battery. ACS Sustain. Chem. Eng..

[24] Gao F., Yan Z., Cai Y., Yang J., Zhong W., Gao Y., Liu S., Li M., Lu L. (2021). 2D leaf-like ZIF-L decorated with
multi-walled carbon nanotubes as electrochemical sensing platform
for sensitively detecting thiabendazole pesticide residues in fruit
samples. Anal. Bioanal. Chem..

[25] Wang T., Kou Z., Mu S., Liu J., He D., Amiinu I. S., Meng W., Zhou K., Luo Z., Chaemchuen S., Verpoort F. (2018). 2D dual-metal zeolitic-imidazolate-framework-(ZIF)-derived
bifunctional air electrodes with ultrahigh electrochemical properties
for rechargeable zinc–air batteries. Adv. Funct. Mater..

[26] Leonowicz M. E., Lawton J. A., Lawton S. L., Rubin M. K. (1994). MCM-22: a molecular
sieve with two independent multidimensional channel systems. Science.

[27] Roth W. J., Nachtigall P., Morris R. E., Cejka J. (2014). Two-dimensional zeolites:
current status and perspectives. Chem. Rev..

[28] Schulman E., Wu W., Liu D. (2020). Two-dimensional
zeolite materials: structural and acidity
properties. Materials.

[29] Choi M., Na K., Kim J., Sakamoto Y., Terasaki O., Ryoo R. (2009). Stable single-unit-cell
nanosheets of zeolite MFI as active and long-lived catalysts. Nature.

[30] Junggeburth S. C., Schwinghammer K., Virdi K. S., Scheu C., Lotsch B. V. (2012). Towards
mesostructured zinc imidazolate frameworks. Chem.—Eur. J..

[31] Junggeburth S. C., Diehl L., Werner S., Duppel V., Sigle W., Lotsch B. V. (2013). Lotsch. B. V.. J. Am. Chem. Soc..

[32] Flügel E. A., Aronson M. T., Junggeburth S. C., Chmelka B. F., Lotsch B. V. (2015). Lotsch.
B. V.. CrystEngComm.

[33] Wan J., Liu D., Xiao H., Rong H., Guan S., Xie F., Wang D., Li Y. (2020). Facet engineering in metal organic
frameworks to improve their electrochemical activity for water oxidation. Chem. Commun..

[34] Xu L., Sun J. (2016). Recent advances in
the synthesis and application of two-dimensional
zeolites. Adv. Energy Mater..

[35] Liu Q., Miao Y., Villalobos L. F., Li S., Chi H. Y., Chen C., Vahdat M. T., Song S., Babu D. J., Hao J., Han Y., Tsapatsis M., Agrawal K. V. (2023). Unit-cell-thick
zeolitic imidazolate framework films for membrane application. Nat. Mater..

[36] Gao X., Chorover J. (2010). Adsorption of sodium
dodecyl sulfate (SDS) at ZnSe
and α-Fe2O3 surfaces: Combining infrared spectroscopy and batch
uptake studies. J. Colloid Interface Sci..

[37] Zhao C. L., Holl Y., Pith T., Lambla M. (1987). FTIR-ATR spectroscopic
determination of the distribution of surfactants in latex films. Colloid Polym. Sci..

[38] Sheldrick G. M. (2008). A Short
History of SHELX. Acta Crystallogr. A.

[39] Sheldrick G. M. (2015). Crystal
Structure Refinement with SHELXL. Acta Crystallogr.
Sect. C Struct. Chem..

[40] Marler B., Gies H. (2012). Hydrous layer silicates
as precursors for zeolites obtained through
topotactic condensation: a review. Eur. J. Mineral..

[41] Chen Y., Huang S., Wang X., Zhang L., Wu N., Liao F., Wang Y. (2017). Synthesis and characterization of
a layered silicogermanate PKU-22 and its topotactic condensation to
a three-dimensional STI-type zeolite. Cryst.
Growth Des..

[42] Asakura Y., Takayama R., Shibue T., Kuroda K. (2014). Topotactic Conversion
of β-Helix-Layered Silicate into AST-Type Zeolite through Successive
Interlayer Modifications. Chem.—Eur.
J..

[43] Asakura Y., Osada S., Hosaka N., Terasawa T., Kuroda K. (2014). Optimal topotactic
conversion of layered octosilicate to RWR-type zeolite by separating
the formation stages of interlayer condensation and elimination of
organic guest molecules. Dalton Trans..

[44] Low Z. X., Yao J., Liu Q., He M., Wang Z., Suresh A. K., Bellare J., Wang H. (2014). Crystal transformation
in zeolitic-imidazolate
framework. Cryst. Growth Des..

[45] Dakhchoune M., Duan X., Villalobos L. F., Avalos C. E., Agrawal K. V. (2023). Hydrogen-sieving
zeolitic films by coating zeolite nanosheets on porous polymeric support. J. Membr. Sci..

[46] Duan X., Dakhchoune M., Hao J., Agrawal K. V. (2023). Scalable
Room-Temperature
Synthesis of a Hydrogen-Sieving Zeolitic Membrane on a Polymeric Support. ACS Sustain. Chem. Eng..

[47] Duan X., Kaya P., Chi H. Y., Topuz B., Agrawal K. V. (2023). Fabrication
of ZIF-8 membranes by direct assembly of nanosheets from bottom-up
synthesis growth solution. J. Membr. Sci. Lett..

[48] De
Yoreo J. J., Vekilov P. G. (2003). Principles of crystal nucleation
and growth. Rev. Mineral. Geochem..

[49] Scholes C. A., Smith K. H., Kentish S. E., Stevens G. W. (2010). CO_2_ capture
from pre-combustion processes-Strategies for membrane gas separation. Int. J. Greenhouse Gas Control.

[50] Kwon H. T., Jeong H. K., Lee A. S., An H. S., Lee J. S. (2015). Heteroepitaxially
grown zeolitic imidazolate framework membranes with unprecedented
propylene/propane separation performances. J.
Am. Chem. Soc..

[51] Andres-Garcia E., Oar-Arteta L., Gascon J., Kapteijn F. (2019). ZIF-67 as silver-bullet
in adsorptive propane/propylene separation. Chem. Eng. J..

