# Supplementary material for: Layered ZIFs Using a Surfactant as a Structure Directing Agent
Source: J Am Chem Soc. 2026 Feb 9;148(7):7231–9. doi: 10.1021/jacs.5c18975 (PMC12951464; doi:10.1021/jacs.5c18975)
Supplement: Supplementary file 1 [file ja5c18975_si_001.pdf]

Supporting Information

Layered ZIFs using a surfactant as a structure directing agent

Xuekui Duan[a,+], Shuqing Song[a,+], Céline Besnard[b], Pascal Alexander Schouwink[c], Yueqing Shen[a], Heng-Yu Chi[a], Jian Hao[a], Laura Piveteau[c] and Kumar Varoon Agrawal[a,]*

[a]*Laboratory of Advanced Separations (LAS), École Polytechnique Fédérale de Lausanne (EPFL), CH-1951 Sion, Switzerland*

[b]*Laboratoire de cristallographie, Ecole de Physique, University of Geneva, 24 Quai E. Ansermet, CH-1211 Geneva 4, Switzerland*

[c]*Institut des Sciences et Ingénierie Chimiques (ISIC), École Polytechnique Fédérale de Lausanne (EPFL), CH-1015 Lausanne, Switzerland*

[+]*These authors contributed equally to this work.*

**Corresponding author.*

E-mail: kumar.agrawal@epfl.ch

Method

1.1 Materials

Zinc nitrate hexahydrate ($Zn(NO_3)_2.6H_2O$, 98%), Cobalt nitrate hexahydrate ($Co(NO_3)_2.6H_2O$, 98%), 2-Methylimidazole (HmIm, $C_4H_6N_2$, 99%), Sodium dodecyl sulfate (SDS, $CH_3(CH_2)_{11}OSO_3Na$, ≥98%) were purchased from Sigma-Aldrich. All the chemicals were used without further purification. PBI-AM Fumion® powder was obtained from Fumatech. Stainless-steel mesh (pore size 20 μm, Part number #325X2300TL0014) was obtained from TWP Inc.

Synthesis of ZIF-S-Zn Nanosheets

The synthesis was done at room temperature using aqueous solutions. Typically, an SDS solution was prepared by dissolving 0.045 g SDS in 100 mL ultrapure water (18.2 MΩ·cm) and mixed well by stirring. Then, 0.0813 g $Zn(NO_3)_2.6H_2O$ was added into the SDS solution and the solution was mixed by stirring. In parallel, an HmIm solution was prepared by dissolving 1.6 g HmIm in 100 mL ultrapure water (18.2 MΩ·cm) and mixed by stirring. The molar ratio of the reactants was $Zn(NO_3)_2.6H_2O$: HmIm : SDS = 1 : 72 : 0.57. The two solutions were kept stir at room temperature until homogenous. Next, the SDS solution was quickly added to the HmIm solution. Reaction was carried out at room temperature for up to 4 hours. Samples were taken from the reaction mixture at I hour, 2 hours and 4 hours respectively, and drop-coated on silicon wafer for scanning electron microscopy (SEM) imaging to monitor the reaction process. The optimum reaction time was selected as 4 hours based on the SEM characterization.

Synthesis of ZIF-S-Co Nanosheets

The synthesis of ZIF-S-Co nanosheets was done similarly to the case of ZIF-S-Zn nanosheets. Typically, an SDS solution was prepared by dissolving 0.045 g SDS in 100 mL ultrapure water (18.2 MΩ·cm) and mixed well by stirring. Then, 0.0786 g $Co(NO_3)_2.6H_2O$ was added into the SDS solution and the solution was mixed by stirring. In parallel, an HmIm solution was prepared by dissolving 1.6 g HmIm in 100 mL ultrapure water (18.2 MΩ·cm) and mixed by stirring. The molar ratio of the reactants was $Co(NO_3)_2.6H_2O$: HmIm : SDS = 1 : 72 : 0.57. The two solutions were kept stir at room temperature until homogenous. Next, the SDS solution was quickly added to the

HmIm solution. Reaction was carried out at room temperature for up to 1 hour since the kinetic of this reaction is faster than the case of ZIF-S-Zn. Samples were taken from the reaction mixture at 5 min, 15 min, 30 min and 1 hour respectively, and drop-coated on silicon wafer for scanning electron microscopy (SEM) imaging to monitor the reaction process. The optimum reaction time was selected as 0.5 hours based on the SEM characterization.

MicroED Measurements and Refinement

T = 100 K 3D-ED data were collected on an FEI Tecnai G2 Sphera equipped with a cheetah D detector with accelerating voltage 160 keV from Amsterdam Scientific. The crystals were cooled during the measurement using a Gatan cryoholder, to decrease radiation damage. Continuous rotation of the sample was applied for a maximum total rotation range of 25 degrees for each crystal. Data from 17 crystals were processed and merged using DIALS.[1,2] The structure could be solved using ShelXD [3] in the space group $P\bar{4}2_1m$, showing a layered structure. The structure was refined in ShelXL.[4]

Room temperature 3D-ED data were collected on an ELDICO ED-1 device (ELDICO Scientific AG) using the software ELDIX.[5] The device is equipped with a LaB_6 electron source operating at an acceleration voltage of 160 kV (λ = 0.02851 Å) and a hybrid-pixel detector (Dectris QUADRO). Diffraction data were recorded in continuous rotation mode with a pseudo-parallel beam of ca. 750 nm diameter. Data were processed using the APEX4 software package.[6] Frames were integrated separately for each crystal, then merged, scaled, and corrected for Lorentz effects, scan speed, background, and absorption using SAINT and SADABS.[7,8] Space group assignment was based on systematic absences, E statistics, and successful refinement of the structure. The structure could be solved using ShelXT in the space group $P\bar{4}2_1m$. The structure was refined with ShelXL in conjunction with ShelXle.[9-11] Least squares refinements were carried out within the kinematic approximation by minimizing $\Sigma w(F_{obs}^2 - F_{calc}^2)^2$ with the ShelXL weighting scheme and using neutral electron scattering factors.[10,12] Non-H atoms were refined with anisotropic displacement parameters. H atoms were placed in calculated positions based on typical distances for neutron diffraction and refined as a rigid rotating group with $U_{iso}(H) = 1.5 \cdot U_{eq}(C)$ for methyl groups and with a standard riding model and $U_{iso}(H) = 1.2 \cdot U_{eq}(C)$ for other groups.

Characterization

Scanning electron microscopy (SEM) images were collected using an FEI Teneo scanning electron microscope with Schottky Field Emission Gun at an acceleration voltage of 2 kV and in-lens detector. Samples were sputter-coated with ~ 5nm thick Iridium layer before imaging to minimize sample charging effect. Transmission electron microscopy (TEM) images were collected using either an FEI Tecnai G2 Spirit transmission electron microscope with a LaB6 source at an acceleration voltage of 120 kV or a Talos F200S G2 Transmission Electron Microscope at an acceleration voltage of 200 kV. Focused-ion beam scanning electron microscopy (FIB-SEM) images were obtained using an FIB-SEM Zeiss CrossBeam 540. X-ray diffraction (XRD) patterns were recorded using a Bruker D8 Discover with CuKα radiation (λ= 1.5406 Å). Thermogravimetric analysis (TGA) was performed with a Perkin Elmer TGA 8000 with heating and cooling rates of 1 °C/min. Differential Scanning Calorimetry (DSC) measurement was performed from 30 to 300 °C in ultra-high-purity air atmosphere on Linseis Simultaneous Thermal Analysis (STA) HP L85 equipped with a differential scanning calorimeter. FTIR (Fourier-transform infrared spectroscopy) measurements were performed on FT-IR-Spectrometer Spectrum Two (PerkinElmer) with an atmospheric vapor compensation feature (an advanced digital filtering algorithm) designed to compensate for CO_2 and H_2O absorptions in real time. Atomic force microscopy (AFM) images were collected using a Bruker MultiMode 8 AFM instrument. Ar adsorption/desorption measurements were performed on an Autosorb 6100 machine at 87 K using a CryoSync. The degasification procedures (120 °C overnight) for the various samples (as-synthesized ZIF-S, calcined ZIF-S and ethanol washed ZIF-S) were the same.

Solid-state NMR spectra were recorded on a 900 MHz Bruker wide bore spectrometer (21.1 T) equipped with an Avance Neo console. ^{1}H Hahn echo NMR spectra and the ^{1}H-^{1}H EXSY spectrum were recorded using a 1.3 mm HCDN quadruple resonance CPMAS probe while spinning the samples at 60 kHz. ^{1}H $\pi/2$ and π pulses of 2.5 and 5 μs respectively were used for both 1D and 2D experiments. Echo delays were set to two rotor periods (33 μs) and mixing time to 10 ms while applying radio-frequency-driven recoupling (RFDR).[13] 16 scans were sufficient to obtain a good signal-to-noise ratio for all the 1D spectra and the EXSY experiment.

The ^{13}C CP and the ^{1}H-^{13}C HETCOR spectra were recorded using a 3.2 mm HCN triple resonance CPMAS probe while spinning the sample at 20 kHz. The spectra were obtained by transferring polarization from ^{1}H to ^{13}C to enhance the signal of the latter or to determine its proximity to protons while applying variable amplitude during the contact time of 1 ms.[14] 16384 transients were cumulated for the ^{13}C CP spectrum and 2048 transients were summed for each slice of the 2D correlation spectrum.

Samples were packed into 1.3 and 3.2 mm zirconia rotors and spun at 60 and 20 kHz respectively. Recovery delays were chosen to be 1.3 times the T_1 of protons, which was extracted from saturation recovery experiments. ^{1}H and ^{13}C chemical shifts were referenced relative to tetramethylsilane.

Membrane Preparation

Porous PBI-AM supports were used for the membrane preparation and they were prepared by the non-solvent induced phase inversion method as reported in literature.[15] Briefly, a polymer dope solution having concentration of 8% (w/w) was prepared by adding commercial PBI-AM Fumion® powder in 1-Methyl-2-pyrrolidinone (NMP) and mixing vigorously using mechanical stir. After stirring for overnight, the dope solution was centrifuged at 40,000 g-force for 3 hours to settle down any undissolved particles. PBI-AM films were casted on stainless-steel metal mesh using a doctor blade casting system. The as-cast films were coagulated in a 60 °C deionized (DI) water bath for 9 hours to allow complete phase inversion and then washed thoroughly with deionized (DI) water before drying. Finally, the supports were dried at room temperature followed by heat treatment at 330 °C for 8 hours with a heating and cooling rate of 0.5 °C /min.

The prepared PBI-AM support was sealed in a home-made filtration cell with Viton® gaskets. The design of the filtration cell was reported in our early report.[16] Membranes were prepared by direct filtration of the nanosheet suspension on PBI-AM supports using a vacuum filtration system. Typically, the as-synthesized ZIF-S-Zn or ZIF-S-Co nanosheets solution was centrifuged at 10,000 rpm for 10 minutes and further sedimented for 24h to settle down thicker nanosheets. Then 3 ml nanosheet suspension was taken from the supernatant containing mostly thinner nanosheets (~5-20 nm). The 3ml nanosheet suspension solution was filtered through the PBI-AM support. Once the filtration was done, the film was dried under vacuum.

Gas permeation

Single gas and mixed gas permeation tests were performed using a homemade permeation setup as reported earlier.[16] In the case of single gas permeation test, 30 mL single gas (H_2, CO_2, N_2, or CH_4) was fed to the membrane through the feed inlet. In the case of mixed gas permeation test, a 50/50 mol% gas mixture was fed to the membrane through the feed inlet. The pressure of feed was maintained at 2 bar. At the permeate side, Ar was used as the sweep gas and the pressure was kept at 1 bar. During test, the chemical potential difference would drive part of the feed gas through the membrane. The permeated gas would be removed by the sweep gas and sent to the mass spectrometer (Hiden Analytical, HPR-20) for a real time analysis. The steady state data was used to calculate the gas concentrations in permeate and further determine the gas permeances and membrane ideal selectivities and separation factors.

Note S1: *Membrane preparation by ethanol washing and secondary growth*

ZIF-S-Co suspension was prepared following the procedures described above. The as-synthesized ZIF-S-Co nanosheets solution was centrifuged at 10,000 rpm for 10 minutes and further sedimented for 24h to settle down thicker nanosheets. Then 3 ml nanosheet suspension was taken from the supernatant containing thinner nanosheets. The 3ml nanosheet suspension solution was filtered through 1 cm diameter PBI-AM support using vacuum filtration. Once the filtration was done, ~ 10 mL ethanol was added and the ethanol was filtered through the deposited ZIF-S-Co film. This in-situ ethanol washing would remove the surfactant in the ZIF-S layers. Finally, the film was left for drying under ambient conditions. After drying, the membrane underwent secondary growth using an ultradilute ZIF-67 precursor solution (4 mM $Co(NO_3)_2$, 32 mM 2-Methylimidazole) at room temperature for 12 h. After 12 h, the membrane was removed from the solution and rinsed in water for a few second. Then, the membrane was left for complete drying in a vacuum oven at 60 °C overnight before permeation tests.

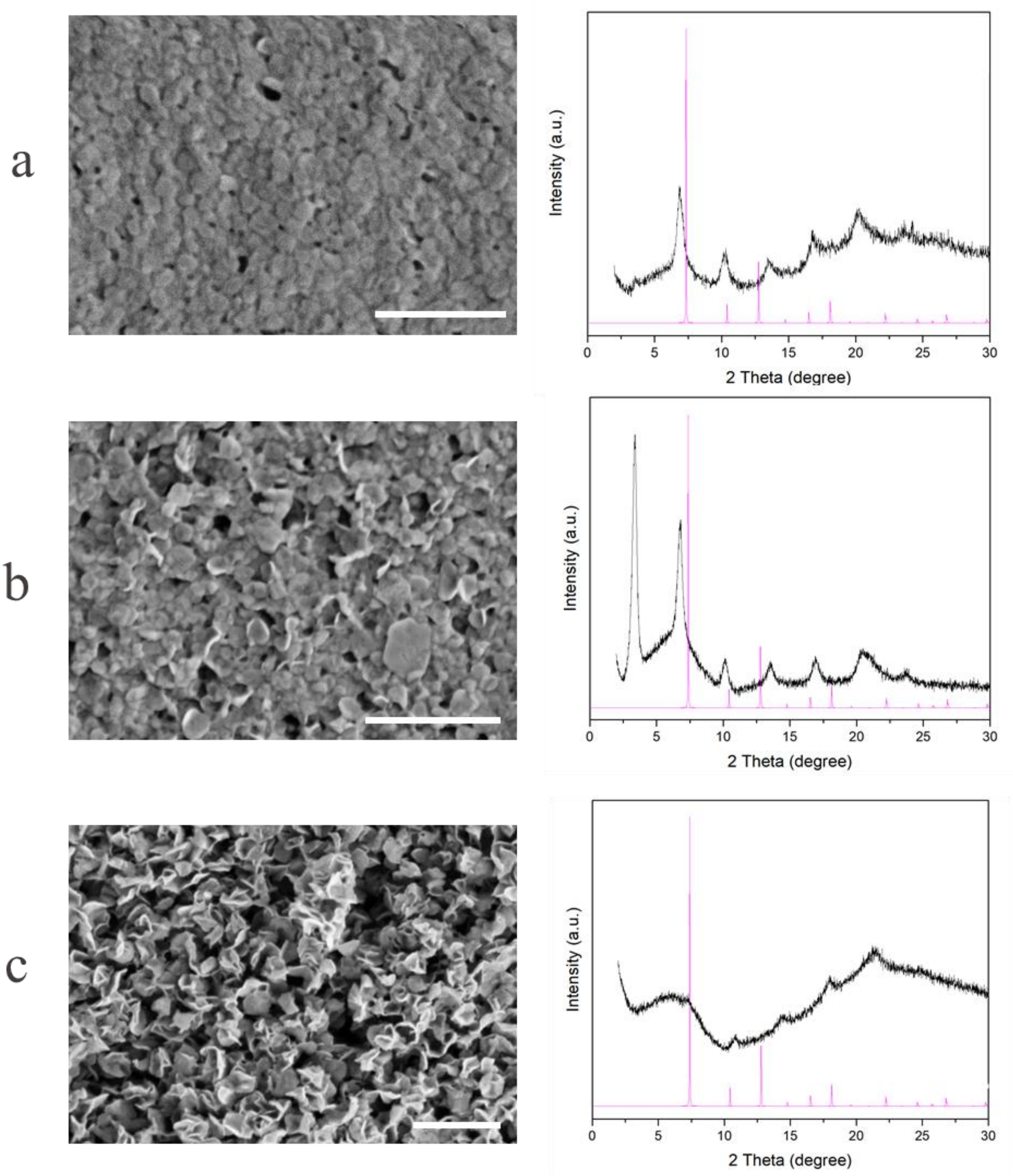

Figure S1: SEM images and corresponding XRD patterns of materials synthesised using Co : HmIm = 2mM : 16 mM and varying concentrations of SDS, a) SDS = 2mM; b) SDS =1mM; c) SDS = 0.5 mM. All reactions were carried out at room temperature for 1 h. Pink XRD patterns represent ZIF-67 reference. Scale bars represent 1 μm.

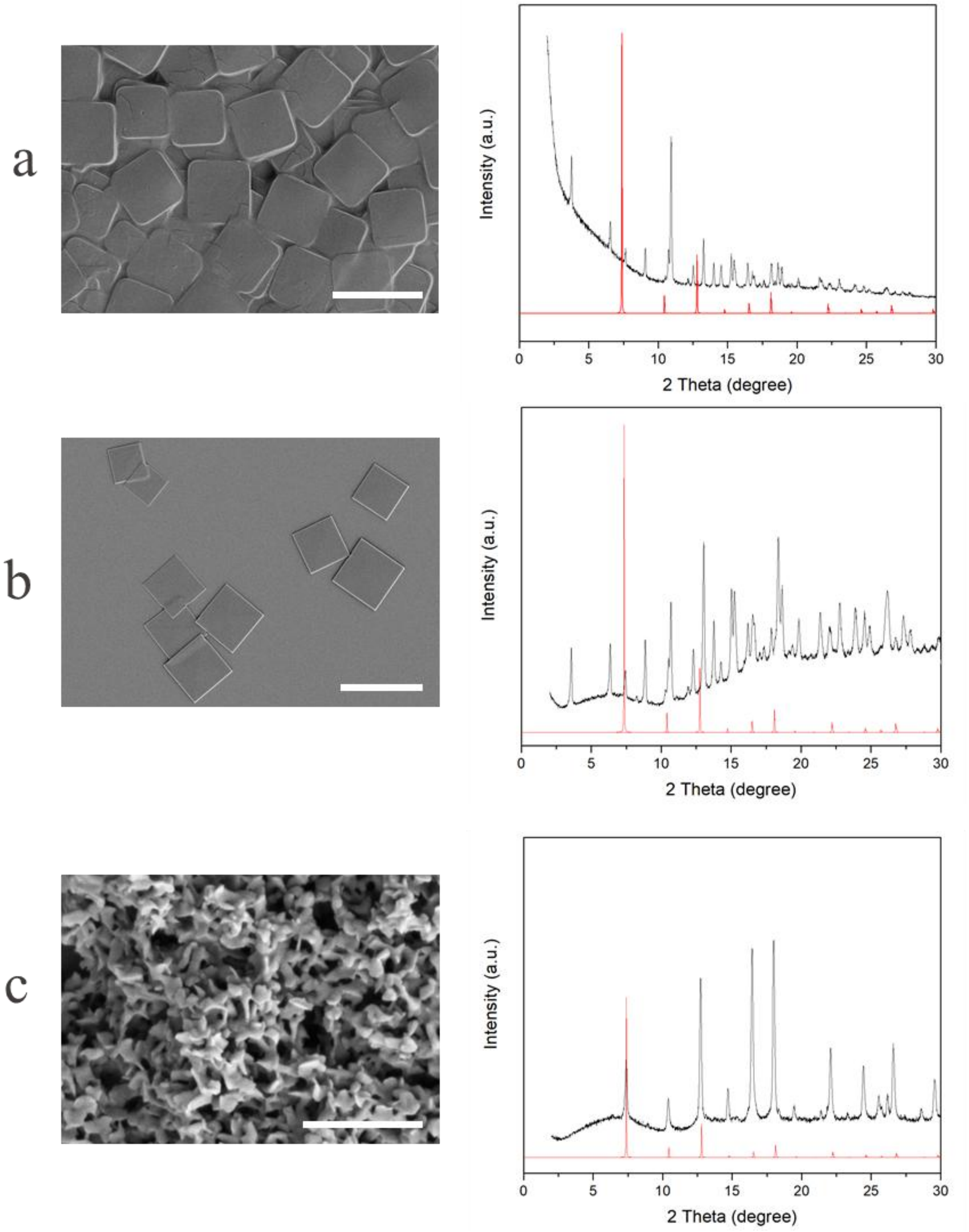

Figure S2: SEM images and corresponding XRD patterns of materials synthesised using Co : HmIm = 1.35mM : 97.44 mM and varying concentrations of SDS, a) SDS = 1.54 mM; b) SDS = 0.77 mM; c) SDS = 0.385 mM. All reactions were carried out at room temperature for 1 h. Red XRD patterns represent ZIF-67 reference. Scale bars in a and b represent 5 μm; scale bar in c represents 1 μm.

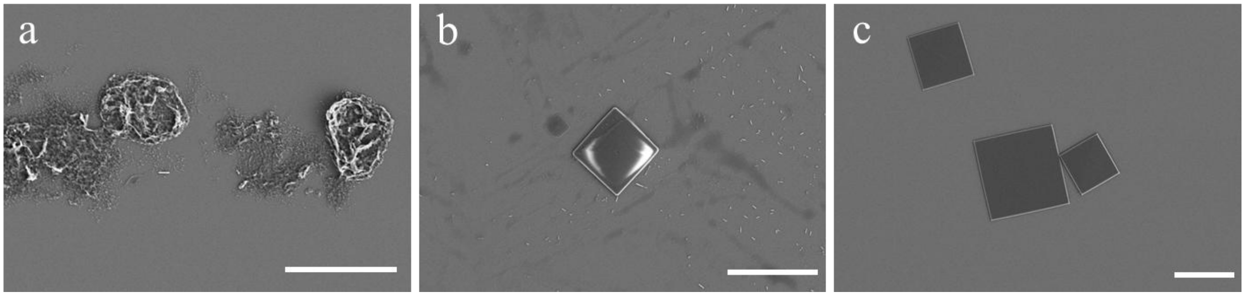

Figure S3: SEM images of ZIF-S-Zn nanosheets growth at room temperatures over synthesis time: a, 1h; b, 2 h; c, 4h. Scale bars represent 5 μm.

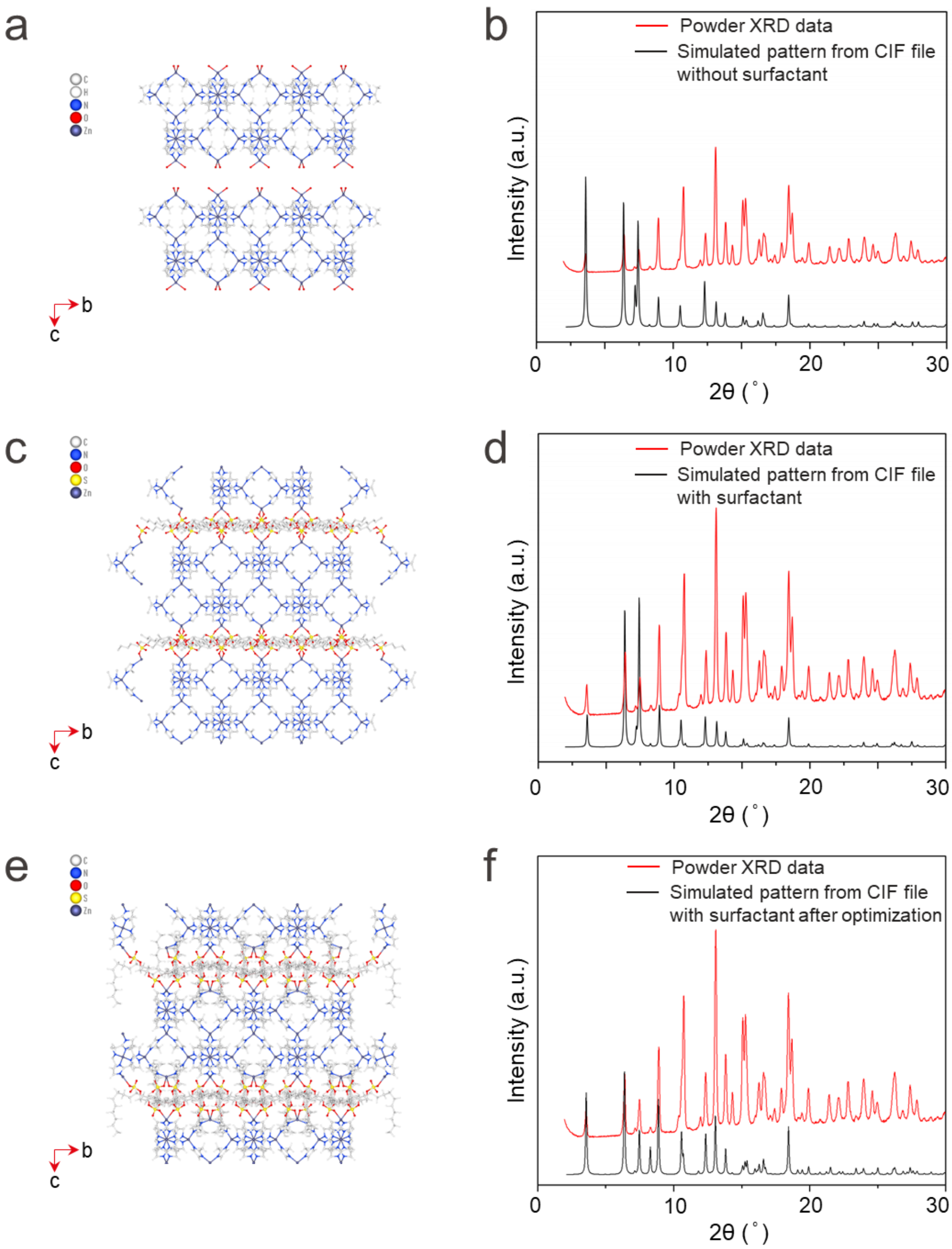

Figure S4: Comparison of different models of ZIF-S material with the corresponding fits between simulated pattern and PXRD pattern. a. ZIF-S structure model without the inclusion of surfactant; b. the corresponding comparison between simulated pattern and PXRD pattern; c. ZIF-S structure model with the inclusion of surfactant; d. the corresponding comparison between simulated pattern and PXRD pattern; e. ZIF-s structure model after optimizing the position of surfactant; f. the corresponding comparison between simulated pattern and PXRD pattern.

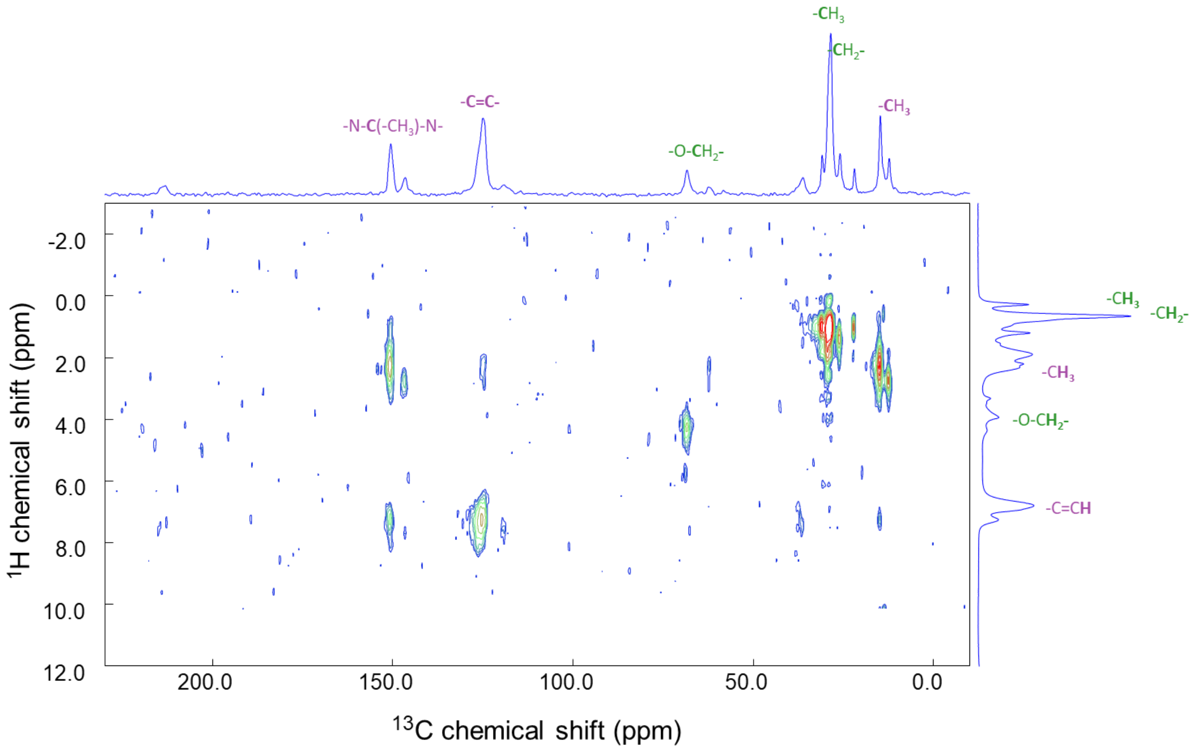

Figure S5: ^{1}H-^{13}C HETCOR solid-state NMR spectrum of the ZIF-S-Zn material.

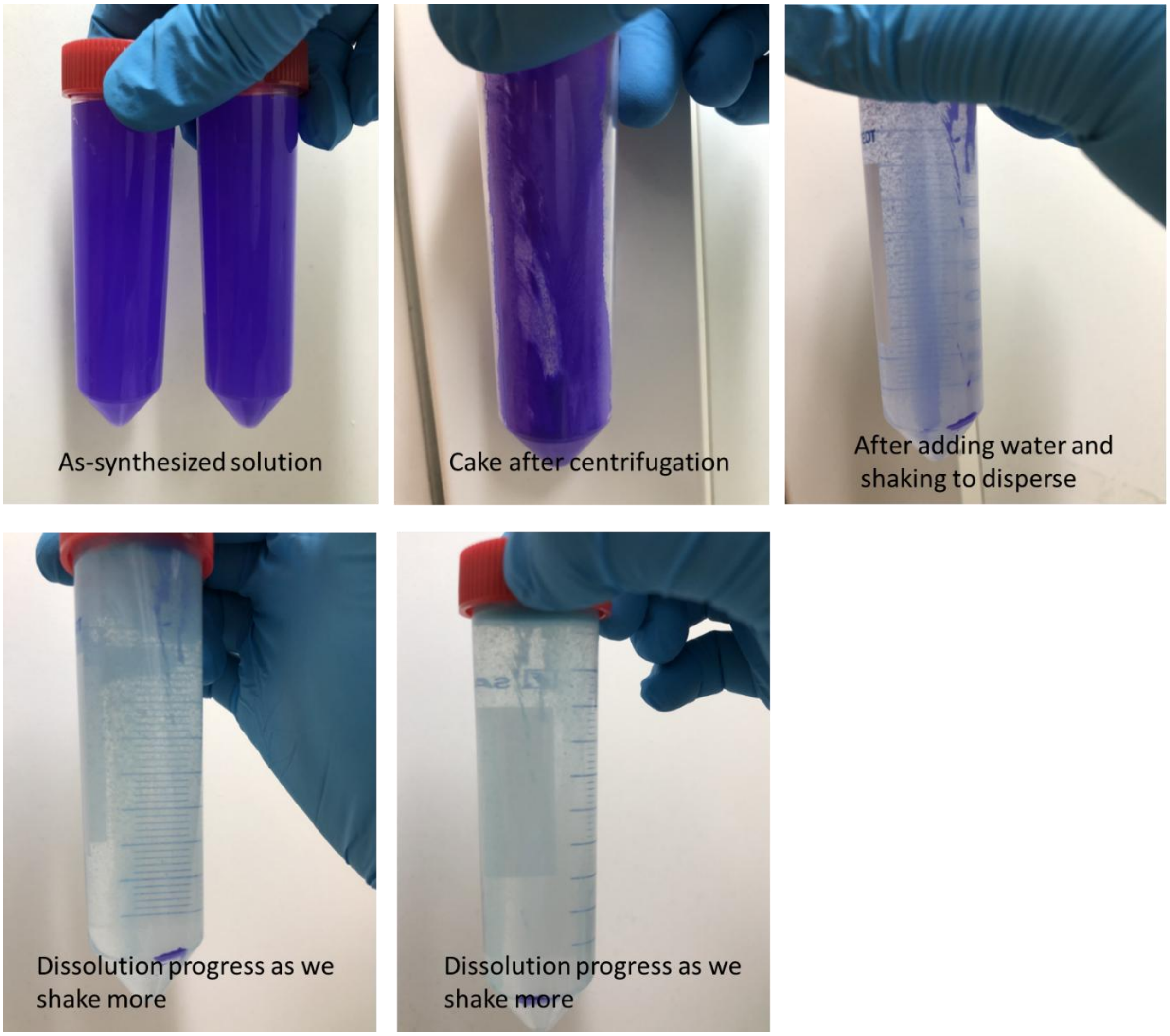

Figure S6: Photographic images illustrating the dissolution of ZIF-S-Co in water during washing processes. ZIF-S-Co was selected for demonstration due to its pronounced color change, which provides a clear visual indication of the phenomenon.

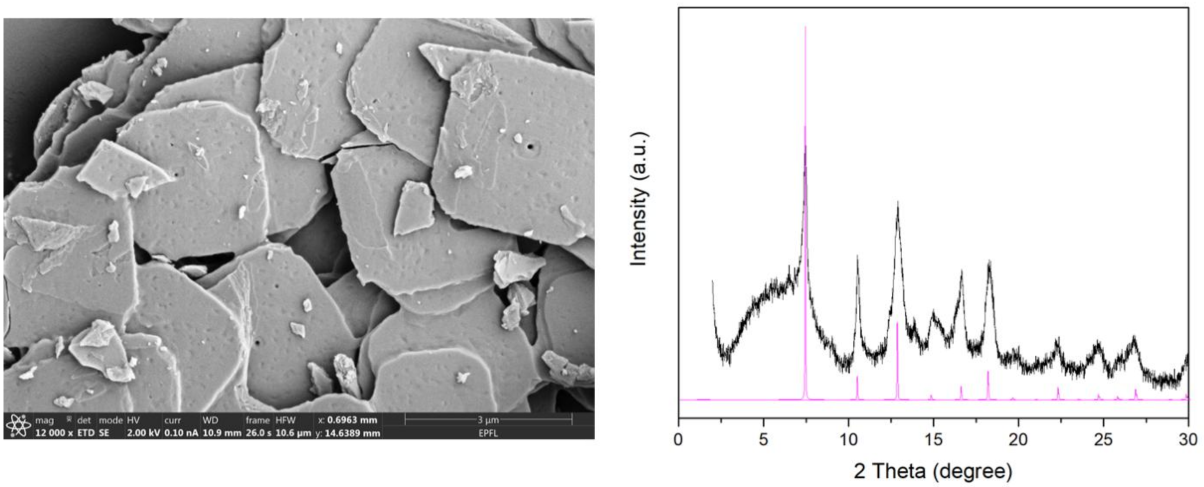

Figure S7: SEM image and the corresponding XRD pattern (black curve) of ZIF-S-Zn after washing with ethanol at room temperature. Pink XRD curve represents ZIF-8 reference.

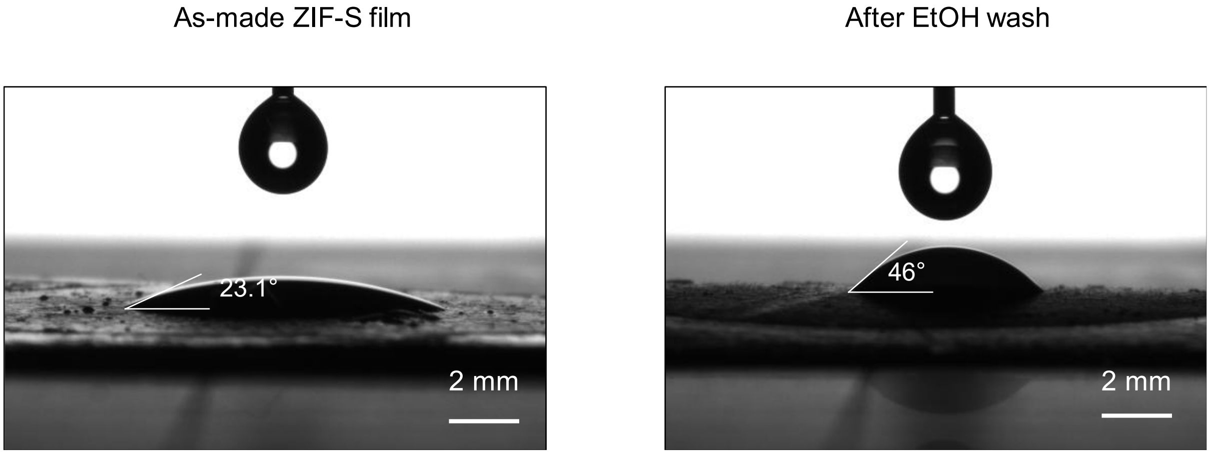

Figure S8: Water contact angle measurement of films made from ZIF-S nanosheets before (left) and after (right) ethanol washing.

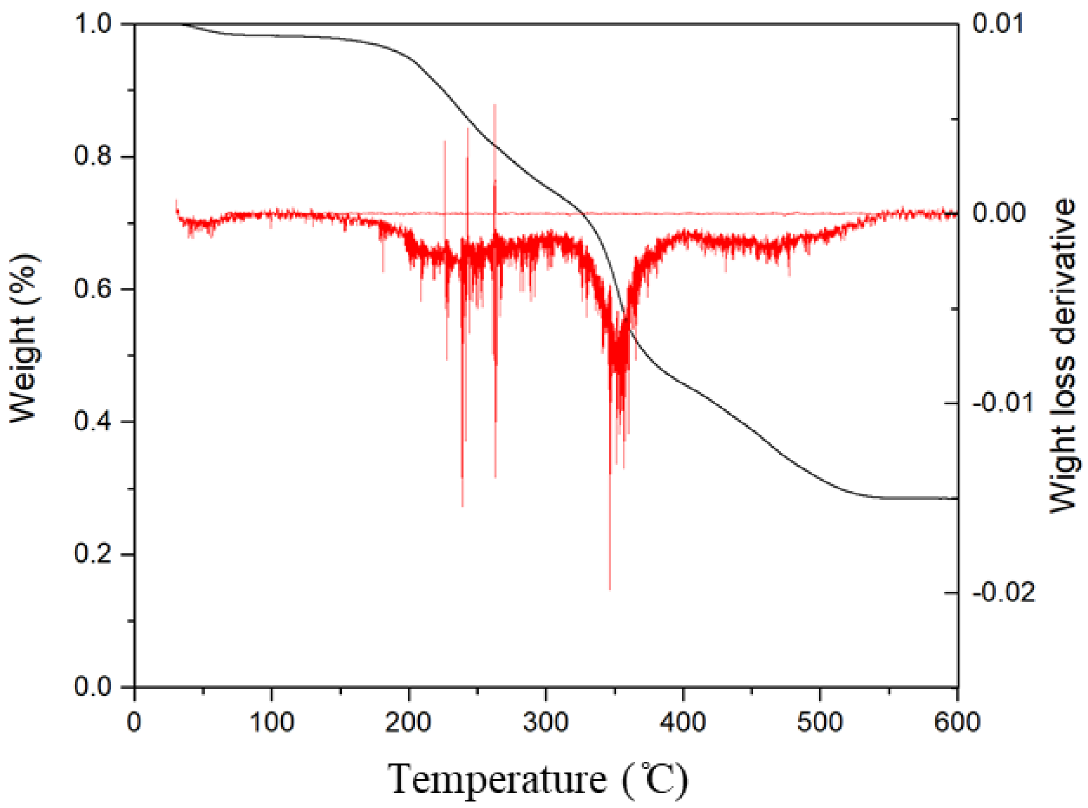

Figure S9: Thermogravimetric analysis (TGA) data from ZIF-S-Zn powder.

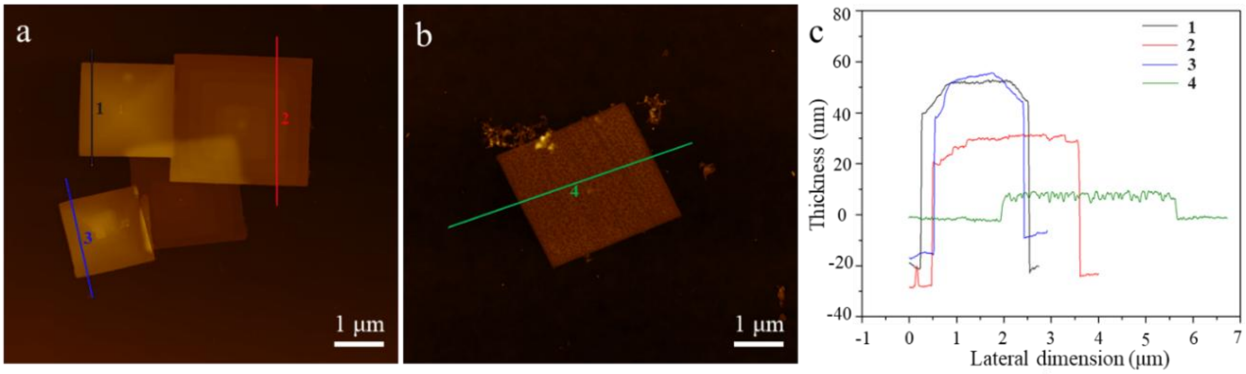

Figure S10: AFM image of the ZIF-S-Zn nanosheets after centrifugation at 10000 rpm for 10 min (a) and after sedimentation for 24 h (b). c, line height profiles of the nanosheets shown in (a) and (b).

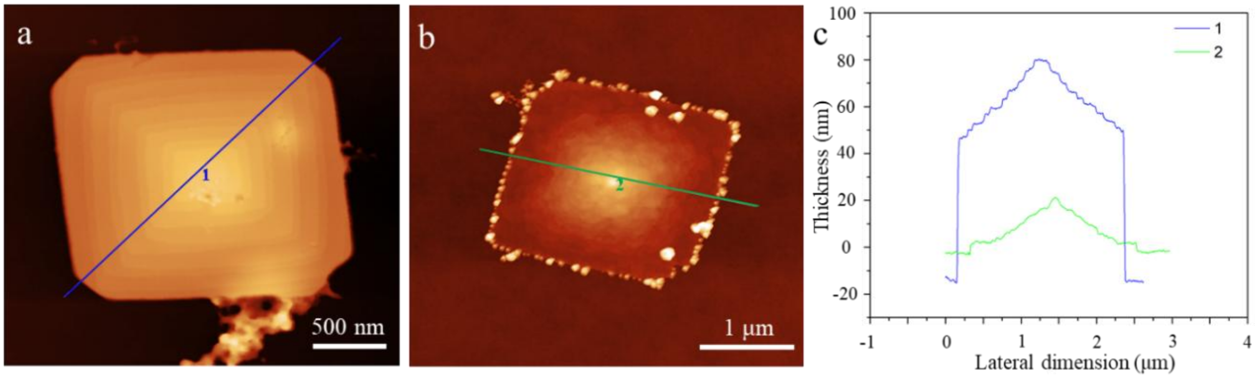

Figure S11: a, AFM image of the ZIF-S-Co nanosheets after centrifuging at 10,000 rpm for 10 min; b, AFM image of the ZIF-S-Co nanosheets after centrifugation at 10,000 rpm for 10 min followed by sedimentation for 24 h; c, the height profiles of the nanosheets shown in a and d along the corresponding lines.

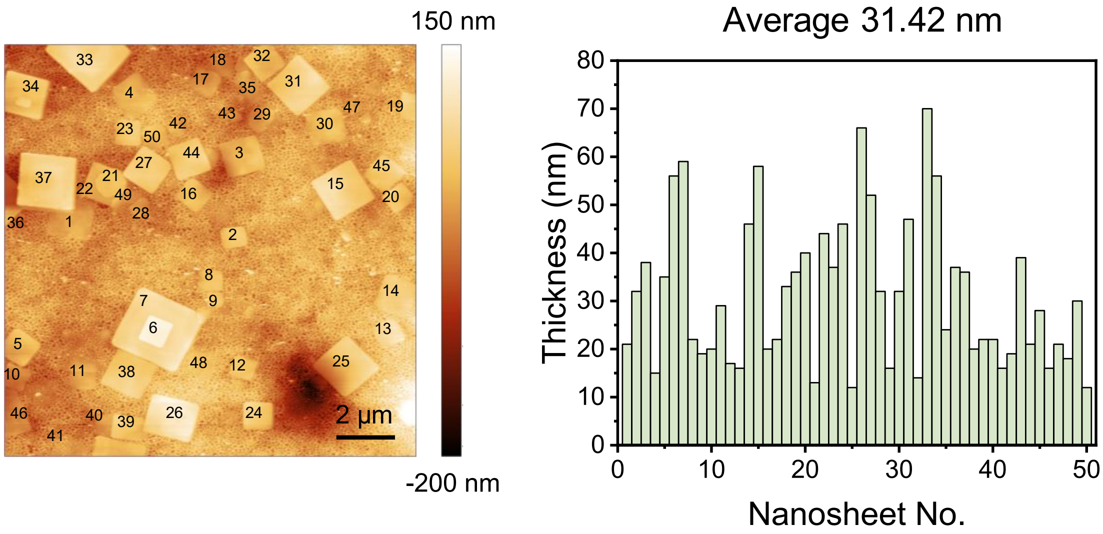

Figure S12: AFM image (left) of the thickness distribution of ZIF-S-Co nanosheets used for membrane preparation and the thicknesses of individual nanosheets (right) marked on the AFM image. Sample was prepared by filtering the nanosheet suspension on smooth porous AAO support.

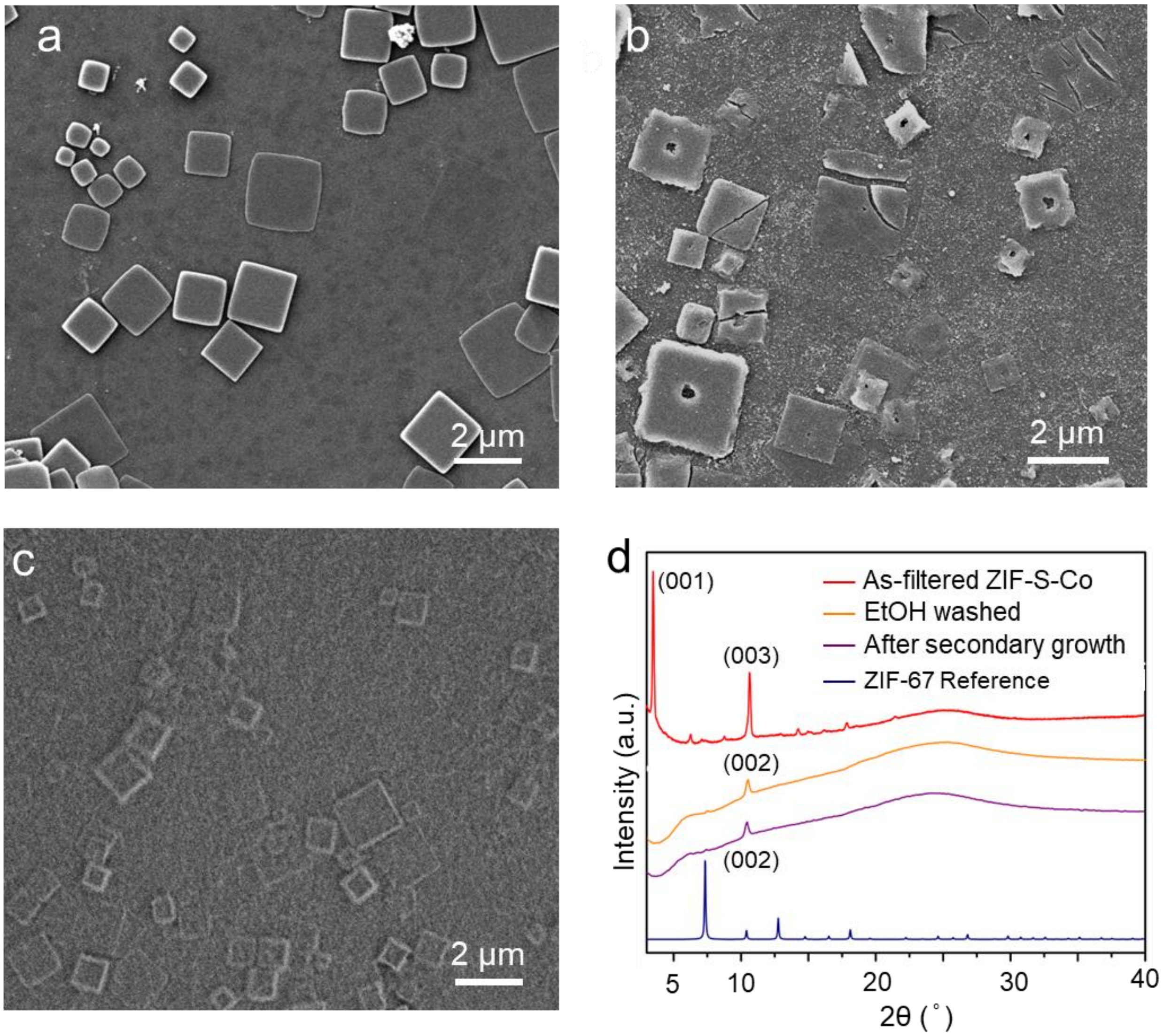

Figure S13: Top-view SEM image of a, as-filtered ZIF-S-Co nanosheet film on PBI support; b, ZIF-S-Co film after ethanol washing; c, ZIF-S-Co film after secondary growth. d, Out-of-plane XRD of the films shown in a-c. The films are highly oriented as the nanosheets lay horizontally on PBI supports.

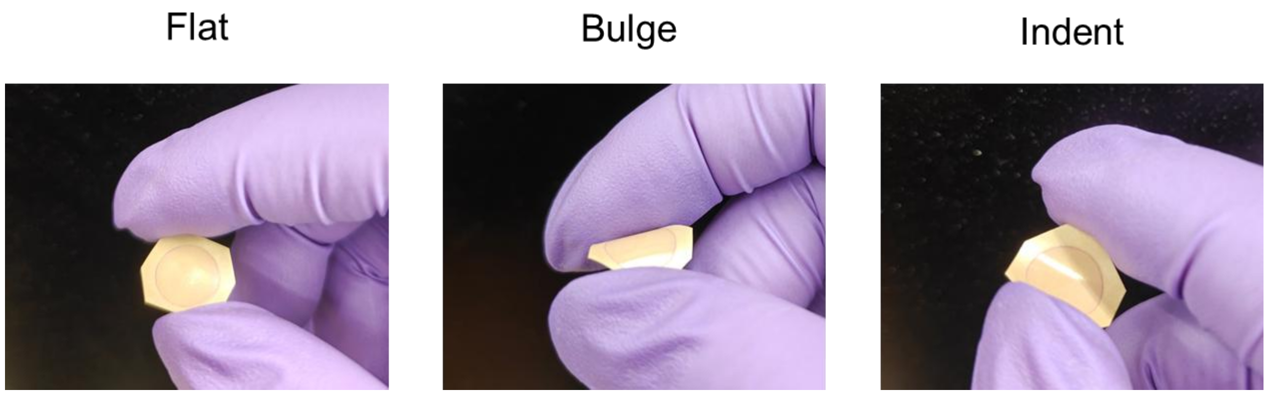

Figure S14: Bending test of the prepared membrane showing the adhesion/mechanical integrity of the membrane. This adhesion/mechanical integrity originate from the bonding between ZIF and PBI substrate due to chemical similarity and hydrogen bonding.

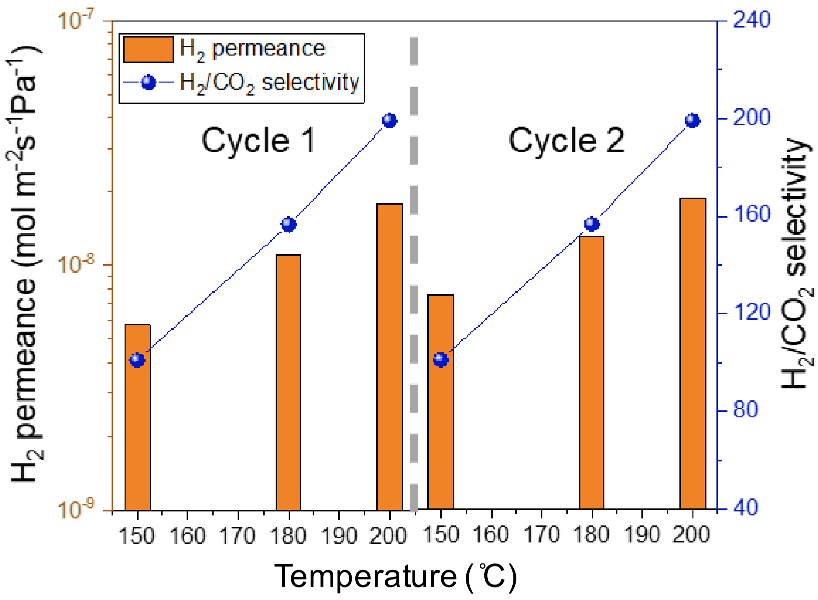

Figure S15: Cycling test of membrane performance for H_2/CO_2 separation. The membrane was first tested at 150 °C, 180 °C and 200 °C in cycle 1; then the membrane was cooled to 150 °C and tested again at 150 °C, 180 °C and 200 °C in cycle 2.

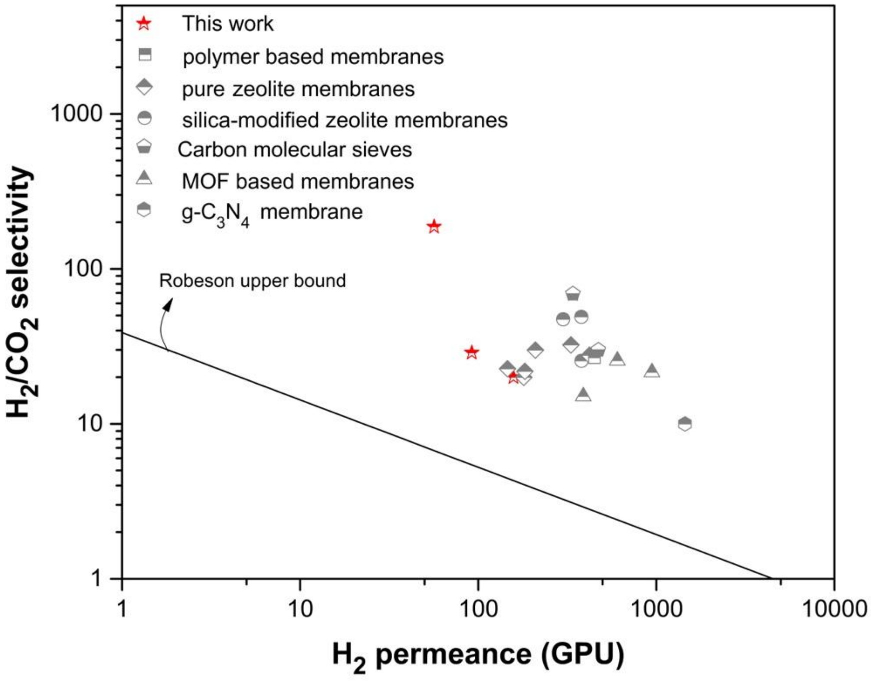

Figure S16: Comparison of the ZIF-S-Co nanosheet membrane prepared in this work with state-of-the-art membranes for H_2/CO_2 separation at temperatures higher than 200 °C. The literature data corresponds to reported H_2/CO_2 performance data from pressurized feed conditions.

Table S1. Literature data of the H_2/CO_2 separation performance from the state-of-the-art membranes at temperatures higher than 200 °C, with pressurized feed.

Membrane	Feed	Pressure	T (°C)	H_2 (GPU)	H_2/CO_2	Ref.
PBI/ZIF-8	Pure	2.5 barg	230	450	27	[17]
B-ZSM-5 silylated	50:50	1.38 barg	400	300	47	[18]
MFI bilayer (with silica)	50:50	1 barg	450	383	25.3	[19]
RUB-15 on AAO	50:50	1 barg	250	211	29.7	[20]
				184	21.7	
			300	424	27.5	
				147	22.5	
				334	32.1	
ZIF/GO	50:50	1 barg	250	388	14.9	[21]
g-C_3N_4	Pure	2 barg	250	1450	10	[22]
RUB-15 on PBI support	50:50	1-3.3 barg	250	180	20	[16]
Hybrid silica	mixture	5 barg	250	383	49.2	[23]
Novolac resin-based CMS	mixture	12.8 barg	250	478	30	[24]
Catalyst packed CMS	mixture	24 barg	250	344	69	[25]
Ultrathin ZIF-67	50:50	1-3 barg	225	620	25	[26]
	50:50	1 barg	250	970	21	
ZIF-S-Co	50:50	1 barg	225	158	20	This work
	50:50	1 barg	200	92	29	
	50:50	1 barg	200	57	187	

Table S2. Mixture gas separation performance of the ZIF-S-Co nanosheet membrane as reported in Figure 5g of the manuscript. The total feed pressure was 2 bar, and the permeate pressure (sweep gas) was 1 bar.

**Gas pairs**	**Temp. (°C)**	**Permeance ($mol\ m^{-2}\ s^{-1}\ Pa^{-1}$)**		**Separation factor (-)**
		**H_2**	**CO_2**	**H_2/CO_2**
**H_2/CO_2**	150	8.6×10^{-9}	3.8×10^{-10}	22.6
	180	1.98×10^{-8}	8.7×10^{-10}	22.7
	200	3.07×10^{-8}	1.06×10^{-9}	28.8
	225	5.29×10^{-8}	2.68×10^{-9}	19.8
**H_2/CH_4**		**H_2**	**CH_4**	**$H_2/\ CH_4$**
	150	9.4×10^{-9}	2.3×10^{-11}	410.4
	180	2.01×10^{-8}	5.2×10^{-11}	388.5
	200	3.01×10^{-8}	1.0×10^{-10}	299.5
	225	5.18×10^{-8}	3.5×10^{-10}	146.2
**H_2/N_2**		**H_2**	**N_2**	**$H_2/\ N_2$**
	150	9.0×10^{-9}	2.5×10^{-11}	9.0×10^{-9}
	180	2.07×10^{-8}	7.8×10^{-11}	2.07×10^{-8}
	200	3.01×10^{-8}	1.6×10^{-10}	3.01×10^{-8}
	225	5.29×10^{-8}	4.9×10^{-10}	107.3

Table S3. Summary of mixture gas permeation data for three ZIF-S-Co nanosheet membranes prepared in the same way (by vacuum filtration) demonstrating the reproducibility of membrane preparation. The measurements were conducted at 200 ºC. The total feed pressure was 2 bar and permeate pressure (sweep gas) was 1 bar.

Membrane 1		Membrane 2		Membrane 3	
H_2 Permeance (mol m^{-2} s^{-1} Pa^{-1})	H_2/CO_2 separation factor (-)	H_2 Permeance (mol m^{-2} s^{-1} Pa^{-1})	H_2/CO_2 separation factor (-)	H_2 permeance (mol m^{-2} s^{-1} Pa^{-1})	H_2/CO_2 separation factor (-)
3.07×10^{-8}	28.8	6.74×10^{-8}	15.8	1.79×10^{-8}	27.5

Membrane 1		Membrane 2		Membrane 3	
H_2 Permeance (mol m^{-2} s^{-1} Pa^{-1})	H_2/CH_4 separation factor (-)	H_2 Permeance (mol m^{-2} s^{-1} Pa^{-1})	H_2/CH_4 separation factor (-)	H_2 permeance (mol m^{-2} s^{-1} Pa^{-1})	H_2/CH_4 separation factor (-)
3.01×10^{-8}	299.5	6.59×10^{-8}	157.7	1.87×10^{-8}	504.7

Membrane 1		Membrane 2		Membrane 3	
H_2 Permeance (mol m^{-2} s^{-1} Pa^{-1})	H_2/N_2 separation factor (-)	H_2 Permeance (mol m^{-2} s^{-1} Pa^{-1})	H_2/N_2 separation factor (-)	H_2 permeance (mol m^{-2} s^{-1} Pa^{-1})	H_2/N_2 separation factor (-)
3.01×10^{-8}	185.5	6.59×10^{-8}	147.8	1.87×10^{-8}	407.8

Reference

1. Winter, G.; Beilsten-Edmands, J.; Devenish, N.; Gerstel, M.; Gildea, R. J.; McDonagh, D.; Pascal, E.; Waterman, D. G.; Williams, B. H.; Evans, G. DIALS as a Toolkit. *Protein Sci.* **2022**, *31* (1), 232–250.

2. Clabbers, M. T. B.; Gruene, T.; Parkhurst, J. M.; Abrahams, J. P.; Waterman, D. G. Electron Diffraction Data Processing with DIALS. *Acta Crystallogr, Sect. D: Struct. Biol.* **2018**, *74* (6), 506-518.

3. Sheldrick, G. M. A Short History of SHELX. *Acta Crystallogr. A* **2007**, 64 (1), 112–122.

4. Sheldrick, G. M. Crystal Structure Refinement with SHELXL. *Acta Crystallogr. Sect. C Struct. Chem.* **2015**, 71 (1), 3-8.

5. ELDIX Software Suite, Version 5.4.0; ELDICO Scientific AG: Allschwil, Switzerland, **2024**.

6. Apex Suite of Crystallographic Software, APEX4, Version 2022.1-1; Bruker AXS Inc.: Madison, Wisconsin, USA, **2022**.

7. SAINT, Version 8.40B; Bruker AXS Inc.: Madison, Wisconsin, USA, **2019**.

8. SADABS, Version 2016/2; Bruker AXS Inc.: Madison, Wisconsin, USA, **2016**.

9. Sheldrick, G. M. SHELXT–Integrated space-group and crystal-structure determination. *Acta Crystallogr., Sect. A: Found. Adv.* **2015**, 71(1), 3-8.

10. Sheldrick, G. M. Crystal structure refinement with SHELXL. *Acta Crystallogr., Sect. C: Struct. Chem.* **2015**, 71(1), 3-8.

11. Hübschle, C. B.; Sheldrick, G. M.; Dittrich, B. ShelXle: a Qt graphical user interface for SHELXL. *J. Appl. Cryst.* **2011**, *44*, 1281–1284.

12. Peng, L. M. Electron atomic scattering factors and scattering potentials of crystals. *Micron* **1999**, *30(6)*, 625-648.

13. Shen, M.; Hu, B.; Lafon, O.; Trébosc, J.; Chen, Q.; Amoureux, J. P. Broadband finite-pulse radio-frequency-driven recoupling (fp-RFDR) with (XY8)41 super-cycling for homo-

nuclear correlations in very high magnetic fields at fast and ultra-fast MAS frequencies. *J. Magn. Reson.* **2012,** 223, 107-119.

14. Peersen, O. B.; Wu, X. L.; Kustanovich, I.; Smith, S. O. Variable-amplitude cross-polarization MAS NMR. J. Magn. Reson. A **1993**, 104, 334-339.

15. Dakhchoune, M.; Duan, X.; Villalobos, L. F.; Avalos, C. E.; Agrawal, K. V. Hydrogen-sieving zeolitic films by coating zeolite nanosheets on porous polymeric support. *J. Membr. Sci.* **2023**; 672, 121454.

16. Duan, X.; Dakhchoune, M.; Hao, J.; Agrawal, K. V. Scalable Room-Temperature Synthesis of a Hydrogen-Sieving Zeolitic Membrane on a Polymeric Support. *ACS Sustain. Chem. Eng.* **2023**, 11(21), 8140-8147.

17. Yang, T.; Shi, G. M.; Chung, T. -S. Symmetric and Asymmetric Zeolitic Imidazolate Frameworks (ZIFs)/Polybenzimidazole (PBI) Nanocomposite Membranes for Hydrogen Purification at High Temperatures. *Adv. Energy Mater.* **2012**, 2(11), 1358-1367.

18. Hong, M.; Falconer, J. L.; Noble, R. D. Modification of Zeolite Membranes for H_2 Separation by Catalytic Cracking of Methyldiethoxysilane. *Ind. Eng. Chem.* **2005**, 44(11), 4035-4041.

19. Wang, H.; Lin, Y. S. Synthesis and modification of ZSM-5/silicalite bilayer membrane with improved hydrogen separation performance. *J. Memb. Sci.* **2012**, 396, 128-137.

20. Dakhchoune, M.; Villalobos, L. F.; Semino, R.; Liu, L.; Rezaei, M.; Schouwink, P.; Avalos, C. E.; Baade, P.; Wood, V.; Han, Y.; Ceriotti, M.; Agrawal, K. V. Gas-sieving zeolitic membranes fabricated by condensation of precursor nanosheets. *Nat. Mater.* **2021**, 20(3), 362-369.

21. Huang, A.; Liu, Q.; Wang, N.; Zhu, Y.; Caro, J. Bicontinuous zeolitic imidazolate framework ZIF-8@GO membrane with enhanced hydrogen selectivity. *J. Am. Chem. Soc.* **2014**, 136(42), 14686-14689.

22. Villalobos, L. F.; Vahdat, M. T.; Dakhchoune, M.; Nadizadeh, Z.; Mensi, M.; Oveisi, E.; Campi, D.; Marzari, N.; Agrawal, K. V. Large-scale synthesis of crystalline g-C_3N_4 nanosheets and high-temperature H_2 sieving from assembled films. *Sci. Adv.* **2020**, 6(4), eaay9851.

23. Koutsonikolas, D. E.; Pantoleontos, G.; Karagiannakis, G.; Konstandopoulos, A. G. Development of H_2 selective silica membranes: Performance evaluation through single gas permeation and gas separation tests. *Sep. Purif. Technol.* **2021**, 264, 118432.

24. Parsley, D.; Ciora, R. J.; Flowers, D. L.; Laukaitaus, J.; Chen, A.; Liu, P. K. T.; Yu, J.; Sahimi, M.; Bonsu, A.; Tsotsis, T. T. Field evaluation of carbon molecular sieve membranes for the separation and purification of hydrogen from coal-and biomass-derived syngas. *J. Membr. Sci.* **2014**, 450, 81-92.

25. Cao, M.; Zhao, L.; Xu, D.; Ciora, R.; Liu, P. K. T.; Manousiouthakis, V. I.; Tsotsis, T. T. A carbon molecular sieve membrane-based reactive separation process for pre-combustion CO_2 capture. *J. Membr. Sci.* **2020**, 605, 118028.

26. Duan, X.; Shen, Y.; Agrawal, K. V. Facile Synthesis of 20-nm-Thick ZIF-67 Films for Hydrogen Sieving Using β-$Co(OH)_2$ Precursor Nanosheets. *Angew. Chem. Int. Ed.* **2025**, 64(45), e202516048.
